# Bulges in left-handed G-quadruplexes

**DOI:** 10.1093/nar/gkaa1259

**Published:** 2021-01-27

**Authors:** Poulomi Das, Khac Huy Ngo, Fernaldo Richtia Winnerdy, Arijit Maity, Blaž Bakalar, Yves Mechulam, Emmanuelle Schmitt, Anh Tuân Phan

**Affiliations:** School of Physical and Mathematical Sciences, Nanyang Technological University, Singapore 637371, Singapore; School of Physical and Mathematical Sciences, Nanyang Technological University, Singapore 637371, Singapore; School of Physical and Mathematical Sciences, Nanyang Technological University, Singapore 637371, Singapore; School of Physical and Mathematical Sciences, Nanyang Technological University, Singapore 637371, Singapore; School of Physical and Mathematical Sciences, Nanyang Technological University, Singapore 637371, Singapore; Laboratoire de Biologie Structurale de la Cellule (BIOC), Ecole Polytechnique, CNRS-UMR7654, Institut Polytechnique de Paris, Palaiseau 91128, France; Laboratoire de Biologie Structurale de la Cellule (BIOC), Ecole Polytechnique, CNRS-UMR7654, Institut Polytechnique de Paris, Palaiseau 91128, France; School of Physical and Mathematical Sciences, Nanyang Technological University, Singapore 637371, Singapore; NTU Institute of Structural Biology, Nanyang Technological University, Singapore 636921, Singapore

## Abstract

G-quadruplex (G4) DNA structures with a left-handed backbone progression have unique and conserved structural features. Studies on sequence dependency of the structures revealed the prerequisites and some minimal motifs required for left-handed G4 formation. To extend the boundaries, we explore the adaptability of left-handed G4s towards the existence of bulges. Here we present two X-ray crystal structures and an NMR solution structure of left-handed G4s accommodating one, two and three bulges. Bulges in left-handed G4s show distinct characteristics as compared to those in right-handed G4s. The elucidation of intricate structural details will help in understanding the possible roles and limitations of these unique structures.

## INTRODUCTION

Four guanine bases with Hoogsteen hydrogen bonds arranged symmetrically between themselves can form a planar structure commonly termed as G-tetrad (1). Such G-tetrads could stack on one another, with the help of monovalent cations, to serve as building blocks in non-canonical structures of nucleic acids known as G-quadruplexes (G4s) (2). G4s were found to exist in important genomic regions such as the telomeres, gene promoters, replication initiation sites and 5′-UTRs, which carry out essential cellular processes (3,4). Besides the biological importance of naturally occurring G4s (4), synthetic G4s are of high interest due to their potential applications in therapeutics (5) and nanotechnology (6). Polymorphism is one of the distinct features of G4 structures, allowing their structural elements—such as strand orientations, loops, and glycosidic bonds—to adopt different conformations depending on the environmental conditions (7–26). Higher-order structures can also be formed by stacking and interlocking of multiple G4 units (27–35).

Bulges are important structural elements of G4s, which form as protrusions of bases from the G-tetrad core. Bulges occur due to discontinuities (presence of non-guanine bases) in one or more G-tracts of the G4 forming sequences. Nearly a third of the experimentally detected G4s in the human genome (over 200 000 out of 700 000) could contain bulges (36). Unlike loops which connect two corners of the G-tetrad core, bulges connect two neighbouring guanines of the same G-tract in the G-tetrad core (37–40). Allowing the presence of bulges in the genomic search increases the number of putative G4 sequence drastically, therefore expanding the library of putative G4-forming sequences (39). Bulges are also important in the context of duplexes, as they can be formed due to mismatches and thus get involved in various interactions with other nucleic acids and proteins (41,42) and be implicated in cellular processes (43–45).

Similar to the B-DNA form (right-handed) and Z-DNA form (left-handed) for a duplex DNA, the backbone progression of G4 DNA is also capable of adopting both right- and left-handed configurations (46). Right-handed G4 structures are known for decades and have been studied extensively (47,48). Left-handed G4s, on the other hand, were recently discovered and remain rather unexplored. Left-handed G4s are characterized by the anticlockwise rotation of the backbone in its helical progression. They have several unique and distinct structural features, such as: (i) ability to form stable four-layered and bi-layered dimeric structures, (ii) stacking of thymine loops with the outer tetrads (T-capping) and (iii) the local sugar orientation of each base is almost perpendicular to the overall backbone progression giving rise to the ‘twisted’ backbone (46,49). Note that these structures are formed by natural DNA, which are to be distinguished from the mirror-image G4 structures formed by enantiomeric L-DNA (50,51). However, the circular dichroism (CD) characteristics of left-handed G4s are noticeably similar to that of L-DNA due to their resemblance in base stacking orientation (50,52). Interestingly, the CD spectrum of a new DNA conformation formed by G_4_C_2_ repeats associated with neurodegenerative diseases exhibits similar features, although the structure has not been resolved yet (53). Characterization of intrinsic fluorescence of DNA secondary structures demonstrated enhanced fluorescence of a left-handed G4 (54). Several families of G4 ligands were reported to exhibit enantioselectivity towards right- and left-handed G4s (55,56), while another can induce right-handed G4 conformation from a sequence favouring left-handed G4 (57).

A study on the impact of sequences to the formation of left-handed G4s revealed that the 12-nt GTGGTGGTGGTG motif (previously named *Block2Δ* (49) and termed as *LHG4motif* in this paper) was a minimal sequence capable of forming a dimeric left-handed G4 on its own and convert certain adjacent G-rich sequences, such as (GGT)_4_—a sequence with four G_2_ tracts forming a right-handed parallel G4 on its own—and several other derivatives to left-handed G4s (Supplementary Figure S1) (49). *LHG4motif* was also shown to convert an adjacent antiparallel G4 to a right-handed parallel G4 (Supplementary Figure S1) (49,58). Bioinformatic search revealed high abundance of *LHG4motif* in the human genome, with over 10 000 hits, exhibiting two-order of magnitude enrichment compared to random occurrence of a 12-nt sequence (49). In this study, to understand the bulge formation in left-handed G4s, *LHG4motif* was attached to G-rich sequences with the potential of forming one or multiple bulges. We showed that *LHG4motif* could convert several adjacent sequences to left-handed G4s, including those which were unstructured on their own. We determined the crystal structure of two left-handed G4s containing one and two bulges, respectively. We have also solved the NMR solution structure of a left-handed G4 containing three bulges. Structural analysis revealed differences between bulges in right- and left-handed G4s. The high abundance of *LHG4motif* in the genome and the feasibility of bulged left-handed G4s driven by this motif can increase the chance of left-handed G4 formation in biological systems. Broadening our understanding of left-handed G4s should be useful for studying their biological functions, as well as their applications in engineering DNA nano-structures.

## MATERIALS AND METHODS

### Sample preparation

Unlabelled DNA oligonucleotides were purchased from IDT with standard desalting purification in the scales ranging from 100 nmol to 1 μmol. Sample purity was measured with ESI-MS and was >99%. The site-specific labelled DNA oligonucleotides were chemically synthesized on an ABI 394 DNA synthesizer using reagents from Glen Research and Cambridge Isotope Laboratories. They were purified following the protocol of Glen Research (https://www.glenresearch.com/glen-pak-dna-purification-cartridge.html). The purified DNA samples were further dialyzed against water, 25 mM KCl and water successively. The dialyzed oligonucleotides were frozen and lyophilized. The DNA samples (concentration, 0.1−1 mM) were dissolved in a buffer containing 70 mM KCl and 20 mM KPi (pH 7), 10% D_2_O, 20 μM DSS. The DNA concentration was measured with UV absorption and expressed in strand molarity using the nearest neighbour approximation for the 260 nm molar extinction coefficient of the unfolded species. DNA samples were annealed by heating at 95°C for 5 mins followed by cooling down slowly to room temperature before subjected to any measurement.

### Circular dichroism

Circular dichroism (CD) spectra were recorded on a JASCO-815 spectropolarimeter using 1-cm path length quartz cuvettes at 25°C. Scans were performed from 220 to 320 nm wavelength with a scanning speed of 100 nm/min, 1-nm data pitch, 2-nm bandwidth and 2 s digital integration time (DIT). Ten accumulations were obtained for each measurement, the spectral contribution of the buffer was subtracted using baseline correction, and data were zero-corrected at 320 nm. DNA samples with concentrations of 3–5 μM were dissolved in a buffer containing 70 mM KCl, 20 mM KPi (pH 7), 10% D_2_O, 20 μM DSS. Molar ellipticity of CD spectra was calculated and reported using the DNA concentration derived from the sample absorbance at 260 nm wavelength and the sample extinction coefficient calculated at 260 nm using nearest neighbour approximation for the unfolded sequence.

### UV melting

UV melting experiments were conducted on a JASCO V-650 spectrophotometer. 3–5 μM DNA samples were taken in a cuvette of a pathlength of 1-cm. UV absorption was measured at 295 and 320 nm wavelength at every 0.5°C between 20 and 90°C. Both the heating and cooling were performed at a rate of 0.1°C/min to allow slow unfolding and folding of the DNA, minimizing hysteresis. The data collected at 295 nm were subtracted from those at 320 nm for background correction and further normalized. The melting temperatures (*T*_m_) were determined from the normalized melting curves where the DNA folded fraction was 50%.

### NMR spectroscopy

NMR experiments were performed on a Bruker spectrometer operating at 600 MHz at 25°C. 0.1−1.5 mM DNA samples dissolved in 70 mM KCl, 20 mM KPi (pH 7), 10% D_2_O, 20 μM DSS were used for NMR measurements. Assignments of the imino protons of guanine residues were obtained by ^15^N-filtered experiments using 2% site-specific labelled samples. Assignments of guanine aromatic protons was obtained via long-range through-bond correlation between imino and aromatic protons. Assignments of other protons were determined based on through-bond (TOCSY/COSY) and through-space correlation experiments. Spectra analyses were performed using the Topspin 3.5 (Bruker) and SPARKY 3.1 (59) software.

### NMR structure calculation

#### NOE distance restraints

Inter-proton distances for *3xBulge-LHG4motif* were obtained from NOESY experiments performed in H_2_O (mixing time, 200 ms) and D_2_O (mixing times, 100, 250 and 300 ms). For non-exchangeable protons, the peaks were classified as strong, medium, medium-weak and weak corresponding to the distance restraints of (2.7 ± 0.8), (3.8 ± 0.9), (4.6 ± 1.2) and (5.5 ± 1.7) Å, respectively. Distances from exchangeable protons were classified as strong, medium and weak corresponding to the distance restraints of (4.0 ± 1.2), (4.8 ± 1.4) and (5.5 ± 1.7) Å, respectively. Distances involving thymine methyl protons (H7#) were altered to be directed towards the methyl carbon (C7) with a 0.5 Å looser upper and lower limits as compensation.

#### Dihedral restraints

Dihedral angle restraints were imposed to the dihedral angle formed by O4’–C1’–N9–C4 of guanine residues. *Anti*-guanine residues were restricted to an angle of (240 ± 70)^o^ or (240 ± 40)° for the outer tetrad and inner tetrad guanines, respectively.

#### Hydrogen-bond restraints

Hoogsteen hydrogen bonds between guanines were restrained using H21–N7, N2–N7, H1–O6 and N1–O6 distances, which were set to (2.0 ± 0.2), (2.9 ± 0.3), (2.0 ± 0.2) and (2.9 ± 0.3) Å, respectively.

#### Planarity restraints

Planarity restraints were used for the G1•G4•G8•G12, G2•G6•G10•G14, G16•G19•G22•G25 and G18•G21•G24•G27 tetrads.

#### Distance-geometry simulated annealing

Initial extended conformation of *3xBulge-LHG4motif* sequence was generated using the XPLOR-NIH (60) program by supplying the available standard nucleic acid topology and parameter tables. Each system was then subjected to distance-geometry simulated annealing by incorporating distance, dihedral, hydrogen bond and planarity restraints. One hundred structures were generated and subjected to further refinement.

#### Distance-restrained molecular dynamics refinement

The 100 structures obtained from the simulated annealing step were refined with a distance-restrained molecular dynamics protocol incorporating all distance restraints. The system was heated from 300 to 1000 K in 14 ps and allowed to equilibrate for 6 ps, during which force constants for the distance restraints were kept at 2 kcal mol^−1^ Å^−2^. The force constants for non-exchangeable proton and exchangeable proton restraints were then increased to 16 and 8 kcal mol^−1^ Å^−2^, respectively, in 20 ps before another equilibration at 1000 K for 50 ps. Next, the system was cooled down to 300 K in 42 ps, after which an equilibration was performed for 18 ps. The coordinates were saved every 0.5 ps during the last 10.0 ps and averaged. The average structure obtained was then subjected to minimization until the gradient of energy was less than 0.1 kcal mol^−1^. Dihedral (50 kcal mol^−1^ rad^−2^) and planarity (1 kcal mol^−1^ Å^−2^ for tetrads) restraints were maintained throughout the course of refinement. Ten lowest-energy structures were generated.

### Crystallization

DNA samples of *1xBulge-LHG4motif-TT* and *2xBulge-LHG4motif-TT* were prepared in 100 mM potassium cacodylate buffer (pH 7) at a concentration of ∼0.8 mM. The samples were annealed by heating at 95°C for 5 min followed by slowly cooling down to room temperature. Initial screening for crystallization conditions were done at 24°C using Natrix 1 and 2 sets of reagents (Hampton Research) in a 96-well sitting drop vapor diffusion setup at 1:1 sample to reagent proportions with the help of mosquito^®^ LCP (ttplabtech). Both the sequences produced crystals under multiple conditions. Crystals were flash frozen in liquid nitrogen before data collection. For *1xBulge-LHG4motif-TT* the crystals grown in 0.08 M potassium chloride, 0.04 M sodium cacodylate trihydrate pH 6.0, 55% (v/v)–2-methyl-2,4-pentanediol and 0.012 M spermine tetrahydrochloride were seen to produce the highest resolution diffraction. For the *2xBulge-LHG4motif-TT*, the crystals obtained in 0.08 M potassium chloride, 0.04 M sodium cacodylate trihydrate pH 7.0, 60% (v/v)–2-methyl-2,4-pentanediol and 0.012 M spermine tetrahydrochloride were chosen for the same reason.

### X-ray diffraction data collection and refinement

Crystal diffraction data were collected at PROXIMA 2 beamline for *1xBulge-LHG4motif-TT* and at PROXIMA 1 beamline for *2xBulge-LHG4motif-TT* of SOLEIL synchrotron, France. Native datasets were collected over 360° rotation ranges at 0.1° oscillation range. Data were processed in *P*2_1_ space group using the XDS software package (61). Molecular replacement was done using the *Z-G4* crystal structure (PDB ID: 4U5M) as a search model to obtain initial phases. One and two copies per unit cell were found respectively for *1xBulge-LHG4motif-TT* and *2xBulge-LHG4motif-TT*. The model was iteratively built through cycles of refinement using phenix (62,63) or Refmac5 (ccp4) and manual rebuilding in coot (64,65). Spermines were fitted into the structures using LigandFit function in Phenix package.

## RESULTS AND DISCUSSION

### *LHG4motif* converts various adjacent sequences to left-handed G4s

Previously, *LHG4motif* was shown to convert an adjacent sequence (TGGTGGTGGTGG) or (GGTGGTGGTGG) containing four G_2_ tracts separated by single thymines to a left-handed G4 ((46,49) and data not shown). To explore the bulge formation in left-handed G4s, additional thymines were added in these G_2_ tracts. Four adjacent sequences which potentially can form one or multiple bulges (Supplementary Table S1) were designed as potential bulge-forming motifs wherein thymines were inserted in the G-tracts as the following: (i) second G-tract (T5), named *1xBulge*; (ii) second and third G-tracts (T5, T9), named *2xBulge*; (iii) second, third and fourth G-tracts (T5, T9, T13), named *3xBulge* and (iv) first, second, third and fourth G-tract (T2, T6, T10, T14), named *4xBulge*. It is to be noted that this nomenclature does not indicate the corresponding bulge formation in these sequences individually. The first sequence on its own formed an unidentified structure, as indicated by six peaks observed in the imino proton region (10–12 ppm) of the one-dimensional (1D) ^1^H NMR spectrum; all the other three sequences were essentially unstructured, as no peaks were observed in the imino proton region (Supplementary Figure S2A). The results were also supported by the CD spectroscopy data (Supplementary Figure S2B).

**Figure 1.**
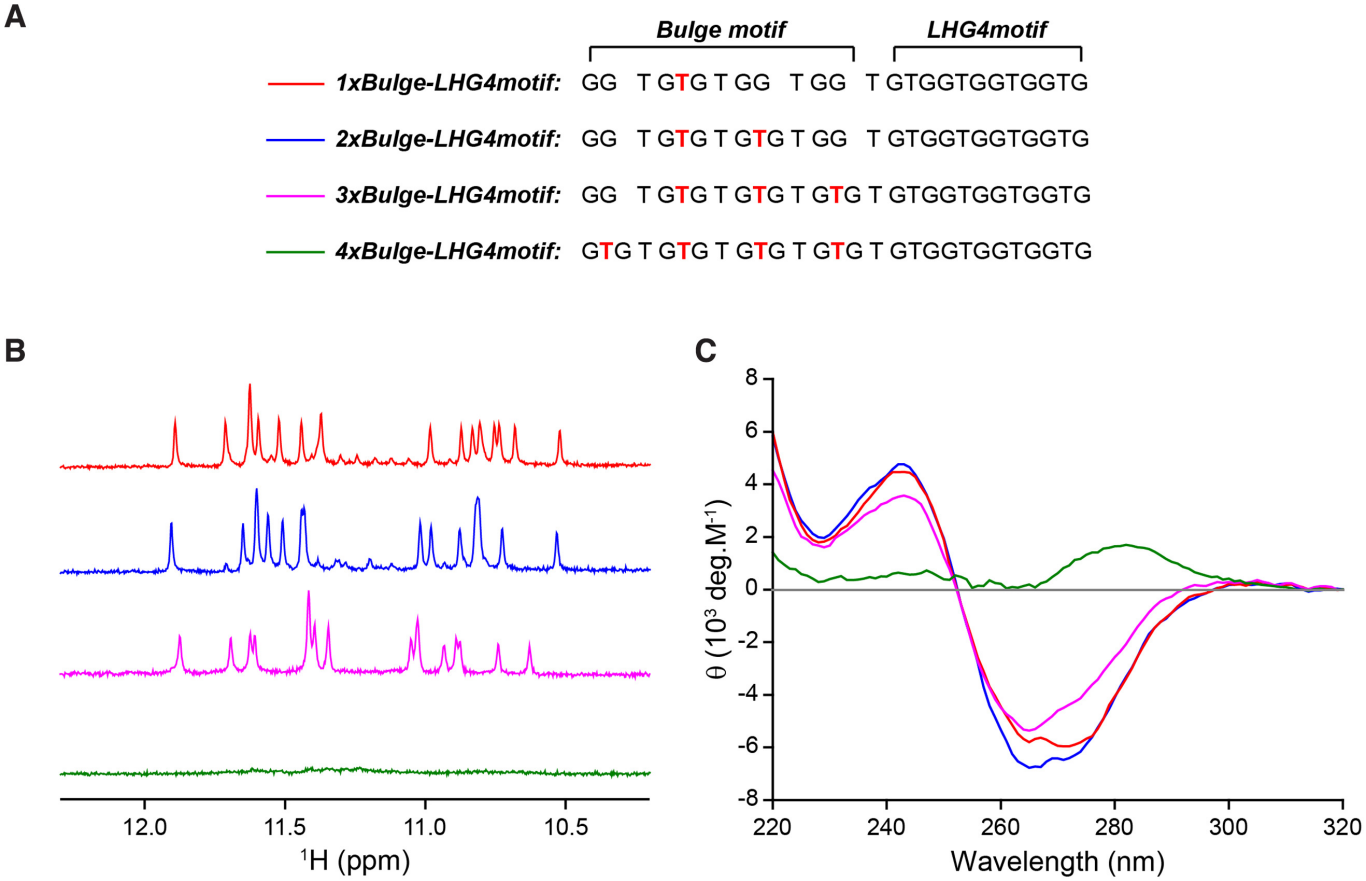
(**A**) Designed sequences attached with *LHG4motif* at their 3′-end. The potential thymine bulges are indicated in red. (**B**) 1D ^1^H NMR and (**C**) CD spectra of left-handed G4s potentially containing one (*1xBulge-LHG4motif*; red), two (*2xBulge-LHG4motif*; blue), three (*1xBulge-LHG4motif*; magenta) and four (*4xBulge-LHG4motif*; green) bulges. The distinct characteristics of left-handed G-quadruplexes can be observed in NMR spectra displaying sixteen peaks divided into two groups of eight peaks, and the molar ellipticity (θ) in the CD spectra possessing crest and trough at 240 and 270 nm, respectively. *4xBulge-LHG4motif* does not form any stable structure.

These four bulge-forming motifs were attached with *LHG4motif* at their 3′-end via a single thymine linker, resulting in sequences named as *1xBulge-LHG4motif, 2xBulge-LHG4motif*, *3xBulge-LHG4motif* and *4xBulge-LHG4motif*, respectively (Figure 1A). Upon combining with *LHG4motif*, the first three sequences exhibited the NMR and CD spectral signatures of left-handed G4s: 1D ^1^H NMR spectra displayed sixteen major imino protons at 10.5–12.0 ppm regrouped into two regions with eight peaks each (Figure 1B); CD spectra showed positive and negative peaks at 240 and 265 nm, respectively (Figure 1C). The fourth sequence, *4xBulge-LHG4motif*, remained unstructured as indicated by NMR and CD spectra (Figure 1B, C). We also investigated the structural driving ability of *LHG4motif* when it is attached to the 5′-end of the designed sequences (Supplementary Table S2). The NMR and CD results suggested that two sequences, *LHG4motif-1xBulge* and *LHG4motif-3xBulge*, but not the sequence *LHG4motif-2xBulge*, were able to form left-handed G4 structures (Supplementary Figure S3). Thus, the structural driving ability of *LHG4motif* was context dependent and appeared to be stronger when attached to the 3′-end rather than the 5′-end of a G-rich sequence. Given the previous observation that a small disruption to the minimal left-handed motif (*LHG4motif*), such as a single thymine addition in a loop, could abolish the left-handed G4 structure (49), we always kept the *LHG4motif* intact in this study.

### Crystal structure of a left-handed G4 with one bulge

**Figure 2.**
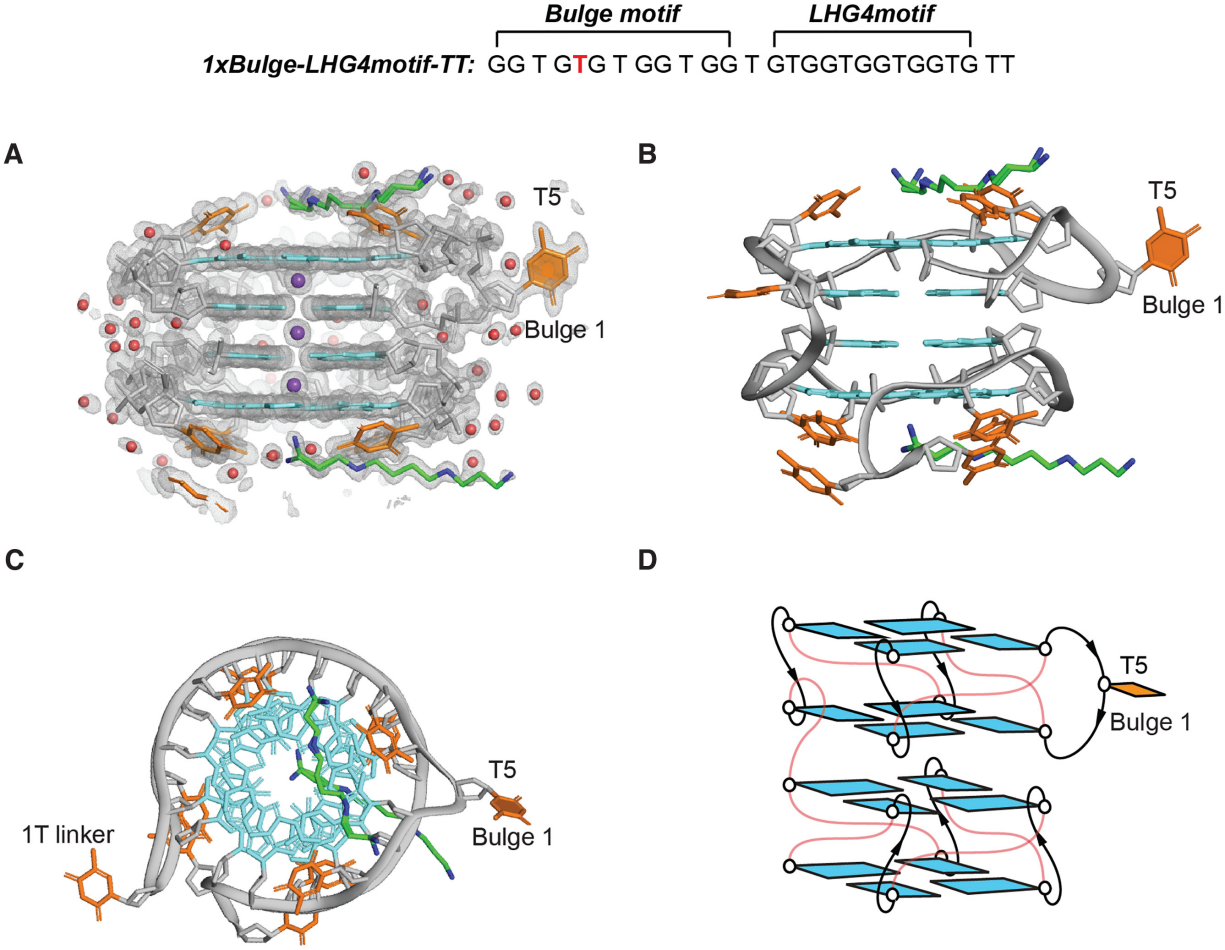
Crystal structure of *1xBulge-LHG4motif-TT*. (**A**) Electron density 2mFo – DFc map of *1xBulge-LHG4motif-TT*. (**B**) Cartoon representation of the crystal structure of *1xBulge-LHG4motif-TT* with the guanine bases coloured in cyan and the thymine bases in orange. Phosphate backbone is shown in gray. Water molecules, potassium ions, spermine carbon and nitrogen atoms are shown in red, purple, green and blue, respectively. (**C**) Top view of the structure showing the T5 bulge in the upper block. (**D**) Schematics of the structure of *1xBulge-LHG4motif-TT* with thymine loops and linkers in red. The single T5 bulge is indicated and highlighted with a filled base in all the panels.

X-ray crystallography was employed to investigate the structure of *1xBulge-LHG4motif*. The sequence *1xBulge-LHG4motif-TT* (Supplementary Table S3) with two additional thymines (TT) at the 3′-end was crystallized. Addition of 3′-end thymines (TT) has improved the crystal packing to provide high-quality X-ray diffraction data. The NMR and CD spectra of *1xBulge-LHG4motif* and *1xBulge-LHG4motif-TT* displayed highly similar spectral characteristics (Supplementary Figure S4), indicating the formation of the same structural conformation by these two sequences. *1xBulge-LHG4motif-TT* was found to crystallize in the *P*2_1_ space group with high-resolution diffraction to 1.18 Å (Table 1). The model fits the electron density excellently as shown in Figure 2A. The crystal structure of *1xBulge-LHG4motif-TT* features a four-layered unimolecular G4 structure separated into two blocks connected by a thymine linker. Each block has parallel left-handed backbone progression similar to the previously reported left-handed G4 structure of *Z-G4* (46). The 5′-5′ stacking interface between guanine bases in successive blocks is in the opposite-polarity mode with partial 5/6-membered ring overlap. The thymines capping of the outer tetrads of the G4 structure (T-capping)—a distinctive feature of left-handed G4s—are also conserved in this structure (46). The upper block has three T-capping residues, while the lower block has four. The thymine residue T5 forming a bulge between the G4 and G6 stacked guanines, projecting out of the G-tetrad core, yielding the first left-handed G4 structure accommodating a bulge (Figure 2B-D).

**Table 1.** Data collection and refinement statistics for X-ray crystal structures of *1xBulge-LHG4motif-TT* and *2xBulge-LHG4motif-TT*

	*1xBulge-LHG4motif-TT*	*2xBulge-LHG4motif-TT*
**Data collection statistics**		
Wavelength (Å)	0.9793	0.9793
Space group	*P*2_1_	*P*2_1_
Cell dimensions (Å/°)	29.2, 40.0, 30.4, 90.0, 115.9, 90.0	29.1, 39.8, 55.0, 90.0, 91.3, 90.0
Resolution range (Å)	26.29–1.182 (1.224–1.182)	29.09–1.296 (1.342–1.296)
Total reflections	139 506 (12 319)	187 246 (14 484)
Unique reflections	20 747 (2060)	31 051 (2861)
Multiplicity	6.7 (6.0)	6.0 (4.9)
Completeness (%)	99.70 (99.95)	94.67 (90.68)
Mean I/sigma(I)	9.00 (1.64)	6.86 (1.07)
Wilson *B*-factor (Å^2^)	8.52	11.81
*R*-merge	0.125 (0.938)	0.164 (1.203)
*R*-meas	0.135 (1.028)	0.180 (1.345)
*R*-pim	0.052 (0.415)	0.073 (0.589)
CC1/2	0.996 (0.5)	0.996 (0.732)
**Refinement statistics**		
Resolution range (Å)	26.29–1.182 (1.224–1.182)	29.09–1.296 (1.342–1.296)
Reflections used in refinement	20 686 (2059)	29 721 (2842)
Reflections used for *R*-free	920 (97)	1433 (135)
*R*-work	0.124 (0.178)	0.184 (0.320)
*R*-free	0.166 (0.204)	0.214 (0.354)
**Number of atoms**		
DNA	569	1178
K^+^	3	7
Na^+^	0	1
Spermine	14	28
Water	158	208
**Average *B*-factor of (Å^2^)**		
DNA	10.5	14.5
K^+^	6.1	13.9
Na^+^	-	12.9
Spermine	22.7	38.2
Water	33.3	26.8
**Rmsd**		
Bond angles, °	2.25	1.04
Bond length, Å	0.018	0.007

Statistics for the highest-resolution shell are shown in parentheses.

The positions of the potassium ions in between the G-tetrads are consistent with those of *Z-G4* and other reported left-handed G4 structures (46,49). The overhang additional 3′-thymines (TT) stack on the capping thymines (Supplementary Figure S5A), while a spermine molecule (from the crystallization solution) stacks over the outer tetrad in each block squeezing itself between the capping thymines (Figure 2). The accommodation of spermine stacking over the tetrads despite the presence of capping thymines establishes the possibility of ligands binding to left-handed G4s.

### Crystal structure of a left-handed G4 with two bulges

**Figure 3.**
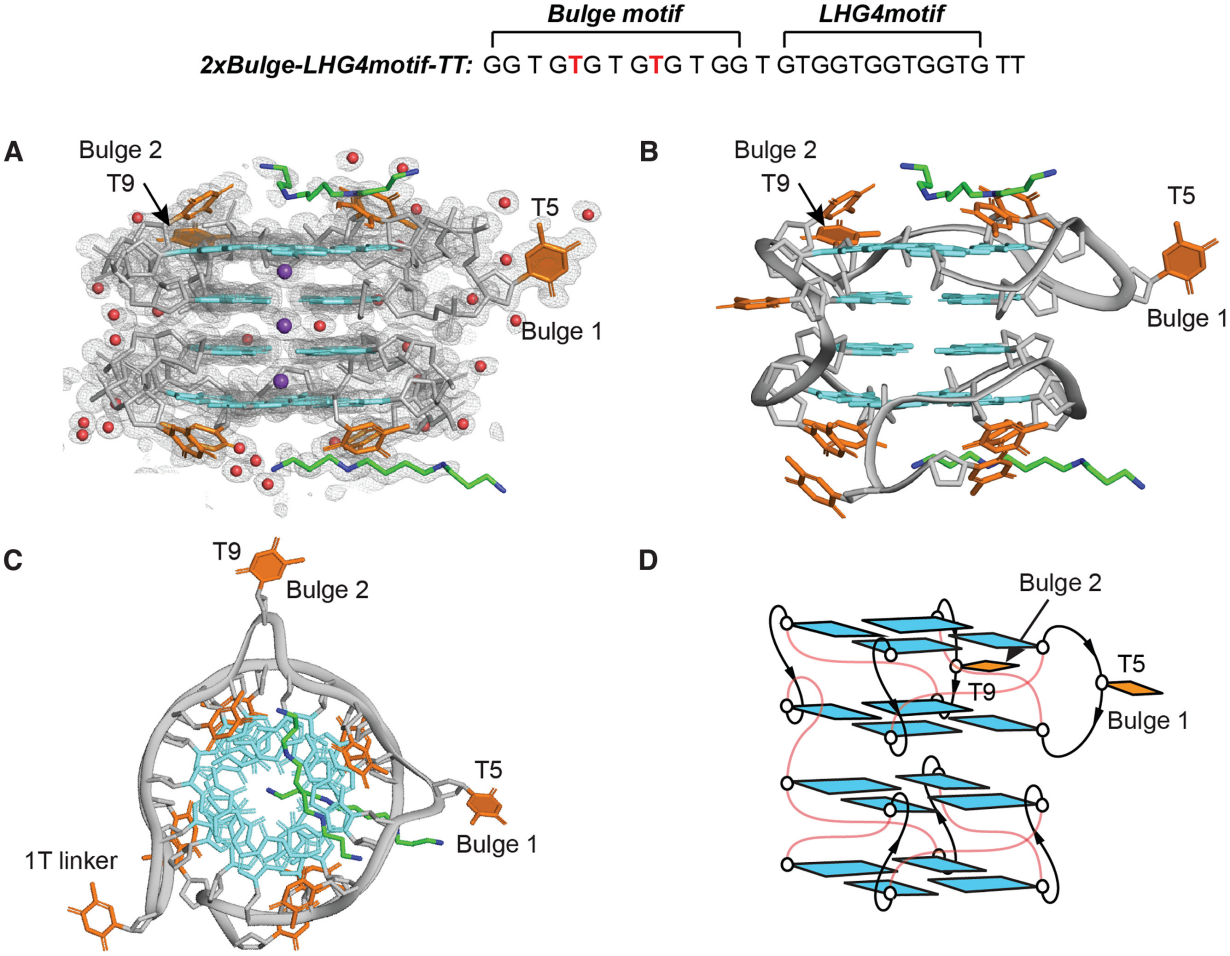
Crystal structure of *2xBulge-LHG4motif-TT*. (**A**) Electron density map of *2xBulge-LHG4motif-TT*. (**B**) Cartoon representation of the crystal structure of *2xBulge-LHG4motif-TT* with the guanine bases coloured in cyan and the thymine bases in orange. Phosphate backbone is shown in gray. Water molecules, potassium ions, sodium ions, spermine carbon and nitrogen atoms are shown in red, purple, yellow, green and blue, respectively. (**C**) Top view of the structure showing the bulges – T5 (Bulge 1) and T9 (Bulge 2) – in the upper block. (**D**) Schematics of the structure of *2xBulge-LHG4motif-TT* with thymine loops and linkers in red. The two bulges (T5, T9) are indicated and highlighted with filled bases.

The sequence *2xBulge-LHG4motif-TT* with two thymines at the 3′-end was crystallized in the *P*2_1_ space group with two molecules in the asymmetric unit (Supplementary Figure S6). The two molecules were related by a translation. Hence, the crystals of *2xBulge-LHG4motif-TT* and*1xBulge-LHG4motif-TT* were packed in a similar fashion. The overall structures are noticeably close to each other with an rmsd of 0.112 Å for all non-H superimposed atoms excluding the bulges. Both crystal structures feature four-layered G4s separated into two blocks connected by one thymine linker. In case of *2xBulge-LHG4motif-TT*, two thymine residues T5 and T9 are projected outward between guanines G4 and G6 as well as G8 and G10, respectively, in the upper block (Figure 3). The overhang 3′-thymines (TT) stack on the capping thymines (Supplementary Figure S5B). A spermine molecule (from the crystallization solution) stacks over the outer tetrad in each block.

The crystal structure of *2xBulge-LHG4motif-TT* also displays three potassium ions coordinated in the central channel of the G4. The two molecules (A and B) in the asymmetric unit are closely similar with an rmsd of 0.132 Å for all non-H superimposed atoms. The molecule B was observed to contain one additional potassium ion in close proximity to thymine T22 and one of the spermine molecule (Supplementary Figure S6). In addition, there is a sodium ion nearby the second bulge T9 in molecule B that interacts with O2 and O4’ of the bulge residue, potentially stabilizing the structure. The sodium ion was modelled to best fit the map, while other molecules such as water or potassium generated significant difference peaks in the Fo – Fc map. The data collection and refinement statistics of the crystal structure *2xBulge-LHG4motif-TT* are summarised in Table 1.

### NMR solution structure of a left-handed G4 with three bulges

As we were unable to obtain good crystals for *3xBulge-LHG4motif* and its derivatives, we proceeded to further characterize the structure of the *3xBulge-LHG4motif* sequence by NMR spectroscopy. The observation of sixteen imino proton peaks supported the formation of a four-layered left-handed G4 by this sequence. These sixteen guanine imino protons were assigned unambiguously by the site-specific 2% ^15^N labeling method (Supplementary Figure S7) (66). Guanine aromatic protons (H8) were assigned via through-bond correlations to imino (H1) protons of the same guanines in a JR-HMBC experiment (Supplementary Figure S8) (66).

To affirm the positions of guanines in the inner and outer tetrads, D_2_O solvent exchange experiment was performed. The imino protons of G1, G4, G8, G12, G18, G21, G24, G27 constituting one group of eight peaks in the downfield chemical shift region disappeared immediately due to exchange with D_2_O solvent, confirming their involvements in the formation of the outer tetrads. The remaining eight peaks of G2, G6, G10, G14, G16, G19, G22, G25 belonging to the other group in the upfield region remained after dissolving the sample in D_2_O (Supplementary Figure S9), consistent with their positions in the inner tetrads, well protected from exchange with solvent. The D_2_O exchange analysis also verified the formation of a four-layered G4 by *3xBulge-LHG4motif*.

**Figure 4.**
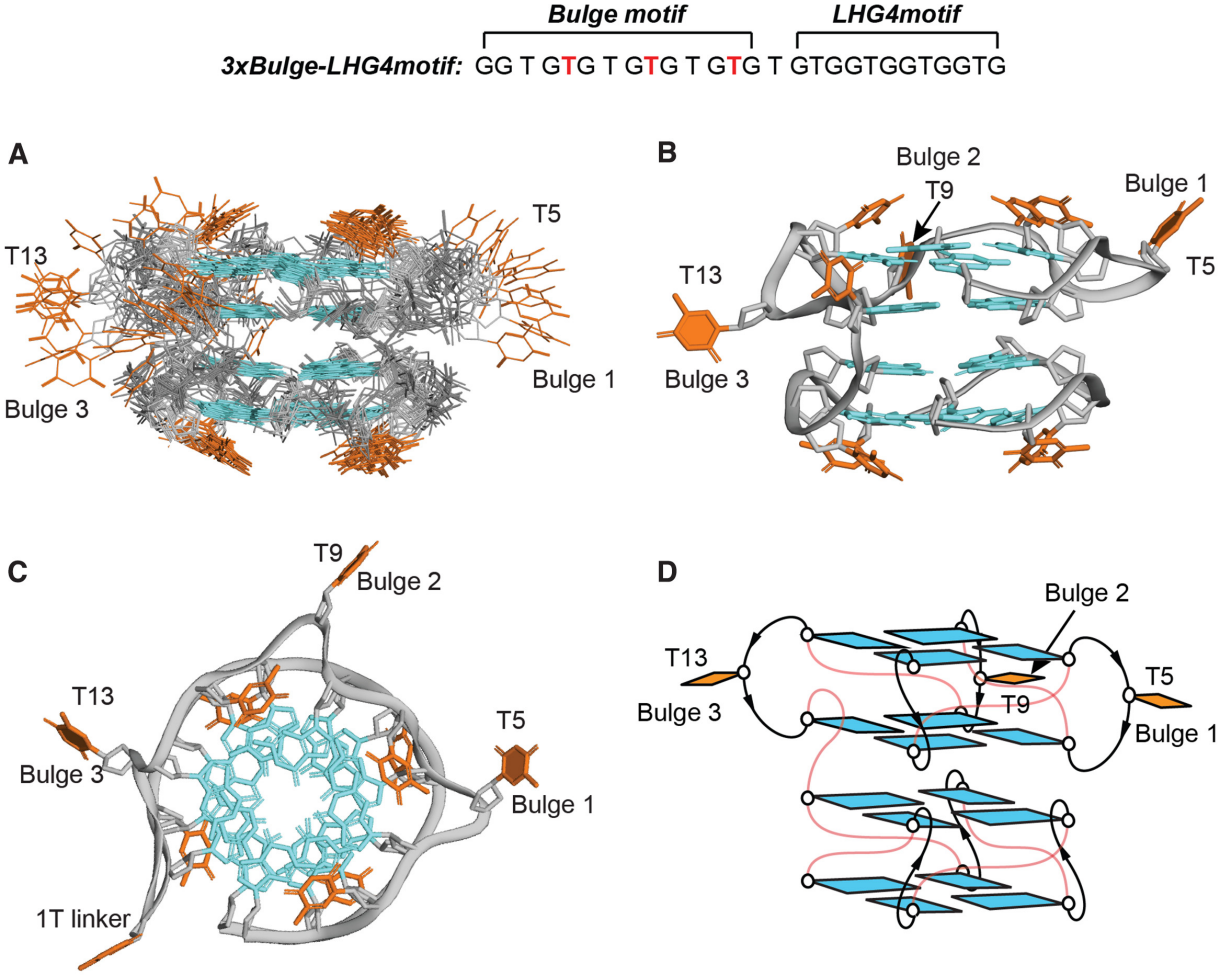
NMR solution structure of *3xBulge-LHG4motif*. (**A**) Ten superimposed lowest-energy structures. (**B**) Cartoon view of a representative structure. Guanine and thymine bases are shown in cyan and orange, respectively. Phosphate backbone is shown in grey. (**C**) Top view of the structure showing the bulges –T5 (Bulge 1), T9 (Bulge 2) and T13 (Bulge 3) – in the upper block. (**D**) Schematics of the structure of *3xBulge-LHG4motif* with thymine loops and linkers in red. The three bulges (T5, T9, T13) are indicated and highlighted with filled bases.

To determine the folding topology of the *3xBulge-LHG4motif*, NOESY experiment (90% H_2_O/10% D_2_O solvent) was performed at a mixing time of 200 ms. Cyclic H1-H8 NOE connectivities between neighbouring guanines in the same tetrads gave rise to the following four-layered topology: G1•G4•G8•G12 (outer), G2•G6•G10•G14 (inner), G16•G19•G22•G25 (inner) and G18•G21•G24•G27 (outer) (Supplementary Figure S10). The glycosidic conformations of all the participating guanine residues were found to be *anti*, deduced from the intensities of intra-residue H8-H1’ cross-peaks in a NOESY spectrum performed in D_2_O at a mixing time of 100 ms, which showed similar moderate intensity for all residues. NOE sequential walk between H8_(*n*)_–H1’_(*n*)_–H8_(*n*+1/*n*+2)_ observed in a NOESY spectrum performed in D_2_O at a mixing time of 300 ms (Supplementary Figure S11) revealed the stacking pattern of the G-tetrads, as well as the thymine loop and bulge arrangements (see caption of Figure S11). The structural schematics of *3xBulge-LHG4motif* is proposed in Figure 4D.

Finally, the NMR solution structure of *3xBulge-LHG4motif* was computed based on NMR constraints obtained from analyses of the NOESY spectra. The structure calculation provided 100 structures based on the distance, angle, hydrogen-bond and planarity constraints, out of which 10 lowest-energy structures were superimposed and presented in Figure 4 with the statistics of the computed structure shown in Table 2. The solution structure of *3xBulge-LHG4motif* revealed a very similar structural characteristics as *1xBulge-LHG4motif* and *2xBulge-LHG4motif* with a four-layered G4 comprising of two blocks connected by a thymine linker. Both the blocks, the lower *LHG4motif* block and the upper block with three bulges, feature parallel left-handed G4 backbone progression. The three bulges T5, T9 and T13 are projecting out between the two stacked guanines in the upper block, while the thymine loops capping at the two ends distinctive to left-handed G4 structures are maintained (Supplementary Figure S12). The three bulges (T5, T9 and T13) and the thymine linker (T15) are not well defined due to lack of constraints. These residues could also be dynamic. While the glycosidic conformations of these residues were not imposed in the structure calculation, diverse conformations were observed among the ensemble of 10 lowest-energy structures, with the majority adopting an *anti*-conformation and a few in *syn*-conformation. The *LHG4motif* block adopts the same structure as reported previously including the thymine loops capping the bottom tetrad.

**Table 2.** Statistics of the computed solution structure of *3xBulge-LHG4motif*

**NMR restraints**		
	**Exchangeable**	**Non-exchangeable**
Distance restraints		
Intra-residue	0	365
Inter-residue	66	163
Other restraints		
Hydrogen bond		64
Dihedral angle		16
**Structure statistics**		
NOE violations		
Number (>2.0 Å)	0.000 ± 0.000	
Deviations from the ideal geometry		
Bond lengths (Å)	0.003 ± 0.000	
Bond angles (°)	0.684 ± 0.006	
Impropers (°)	0.351 ± 0.004	
Pairwise heavy atom RMSD value (Å)		
G-tetrad core	0.636 ± 0.085	
All heavy atoms	2.023 ± 0.357	

### The destabilizing effect of additional bulges: left-handed *versus* right-handed G4s

Previously, simultaneous accommodation of up to three bulges in different positions of a right-handed G4 was demonstrated (39). In that study, G4 structures with more than three bulges could not be formed, coincident with our current study where *4xBulge-LHG4motif* was unable to form any stable structure. The comparison between the costs in the thermal stability upon increasing the number of bulges is summarized (Supplementary Table S4) (39). In the right-handed G4 system, on average, the melting temperature decreased by ∼17°C upon addition of a second bulge in a different G-tract with respect to the first bulge (39). It further decreased by ∼21°C due to the addition of a third bulge in a different G-tract (39). Comparatively, in the current left-handed G4 system, the melting temperature decreased by ∼16°C when the second bulge (T9) was introduced in *2xBulge-LHG4motif* in addition to the first one (T5) in *1xBulge-LHG4motif*. The stability was further decreased by ∼12°C when the third bulge was added in *3xBulge-LHG4motif* (Supplementary Figure S13). Taken together, we found a diminished destabilizing effect of additional bulges in left-handed G4s compared to that of right-handed G4s. The comparison with no-bulge left-handed G4 structures was not attempted due to the presence of more than one conformation in the no-bulge construct.

### Effect of bulges in altering the backbone dihedral angles in the G-tetrad core: left-handed *versus* right-handed G4s

The dihedral angles of left- and right-handed G4 backbones were previously compared, showing significant differences between them (46). Here, we compare the same set of stepwise dihedral angles alterations (*ϵ, ζ, α+1, β+1* and *γ+1*) upon addition of bulges. Specifically, we quantified the dihedral angle adjustments on the core guanine residues on the immediate 5′-side of the bulge (termed ‘previous residue’) as well as the core guanine residue on the immediate 3′-side of the bulge (termed ‘next residue’) in the systems of left- and right-handed G4s. The dihedral angle alterations are compared with the standard set of dihedral angle values of parallel G4s without bulges obtained from selected crystal structures deposited in PDB for both left- (4U5M, 6GZ6) (46,49) and right-handed systems (1KF1, 244D, 352D) (8,67,68).

**Figure 5.**
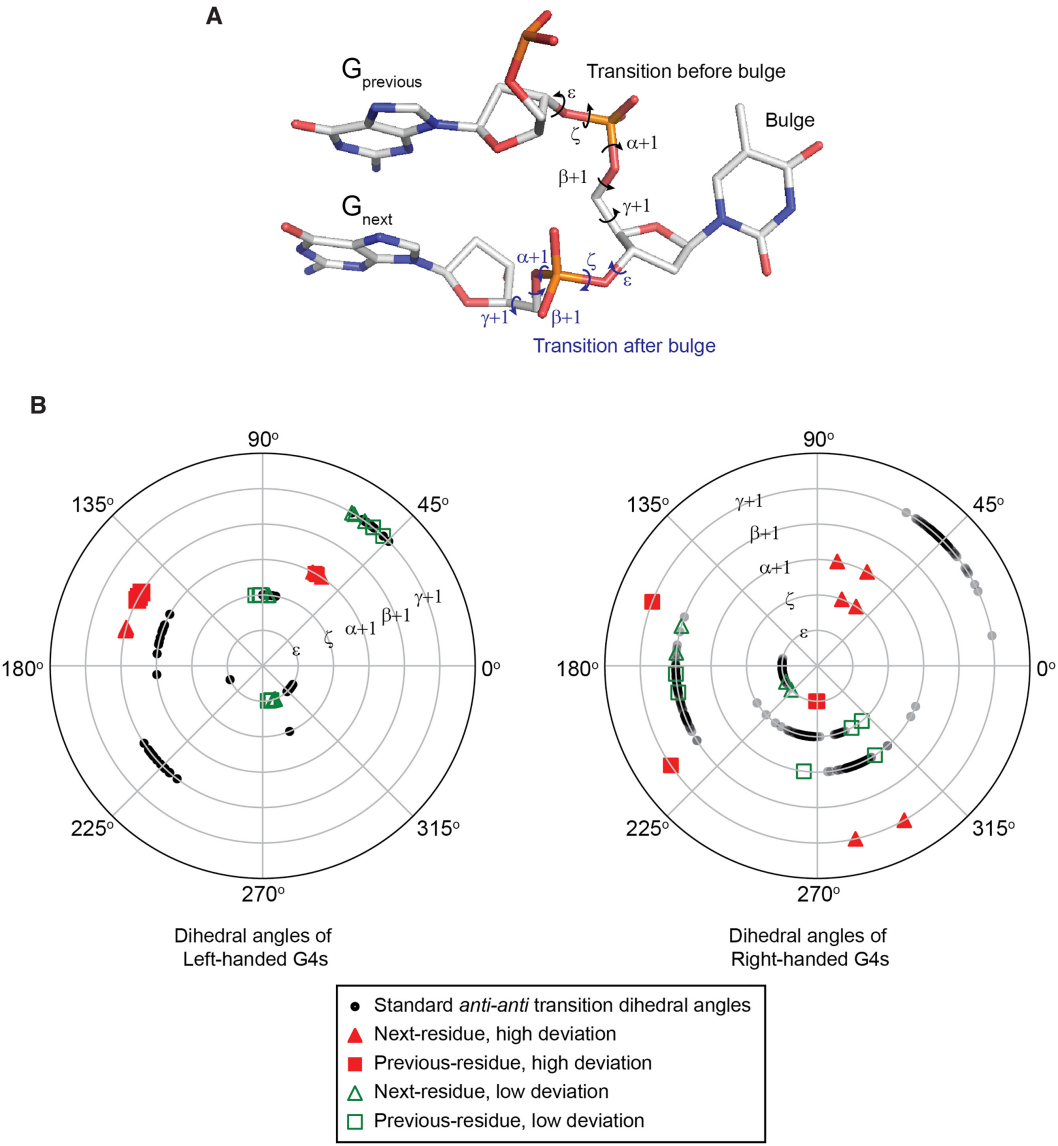
Comparison of dihedral angles between bulged and non-bulged G4s in left- and right-handed systems. (**A**) Schematics of the transitional dihedral angles before (‘previous residue) and after (‘next residue’) the bulge. (**B**) Comparison diagrams of the left- and right-handed G4 dihedral angle values. Black circles indicate standard values (PDB ID: 4U5M, 6GZ6, 1KF1, 244D and 352D) (8,46,49,67,68); solid red triangles and squares indicate high-deviation bulged values; open green triangles and squares indicate low-deviation bulged values (structures of *1xBulge-LHG4motif-TT, 2xBulge-LHG4motif-TT* and PDB ID: 5UA3) (40).

The comparison results are summarized in Figure 5. The standard dihedral angle values are represented in black open circle, solid red symbols represent the dihedral angle values from the bulged systems that were significantly different from the reference values (>60°), whereas open green symbols indicate the dihedral angle values close to the standard values. The introduction of a bulge in a left-handed G4 altered the transition angles *α*+1 and *β*+1 for both the ‘previous residue’ and ‘next residue’ by ∼90° and ∼70° respectively, with all other angles largely unaffected. On the other hand, the bulge in a right-handed G4 affected the angles *ϵ* and *γ*+1 for the ‘previous residue’ by ∼90° and ∼135° respectively, as well as the angles *ζ, α*+1 and *γ*+1 for the ‘next residue’ by ∼180°, ∼135° and ∼120° respectively. We conclude that the bulges in left-handed G4s are less disruptive to the neighbouring residues in terms of dihedral angle values than their right-handed counterparts. The data of bulged left-handed G4s were taken from the crystal structures of *1xBulge-LHG4motif-TT* and *2xBulge-LHG4motif-TT* in this paper, while the bulged right-handed G4 data were taken from the deposited crystal structure 5UA3 (40).

### Effect of bulges in altering the sugar puckering in the G-tetrad core: left-handed *versus* right-handed G4s

**Figure 6.**
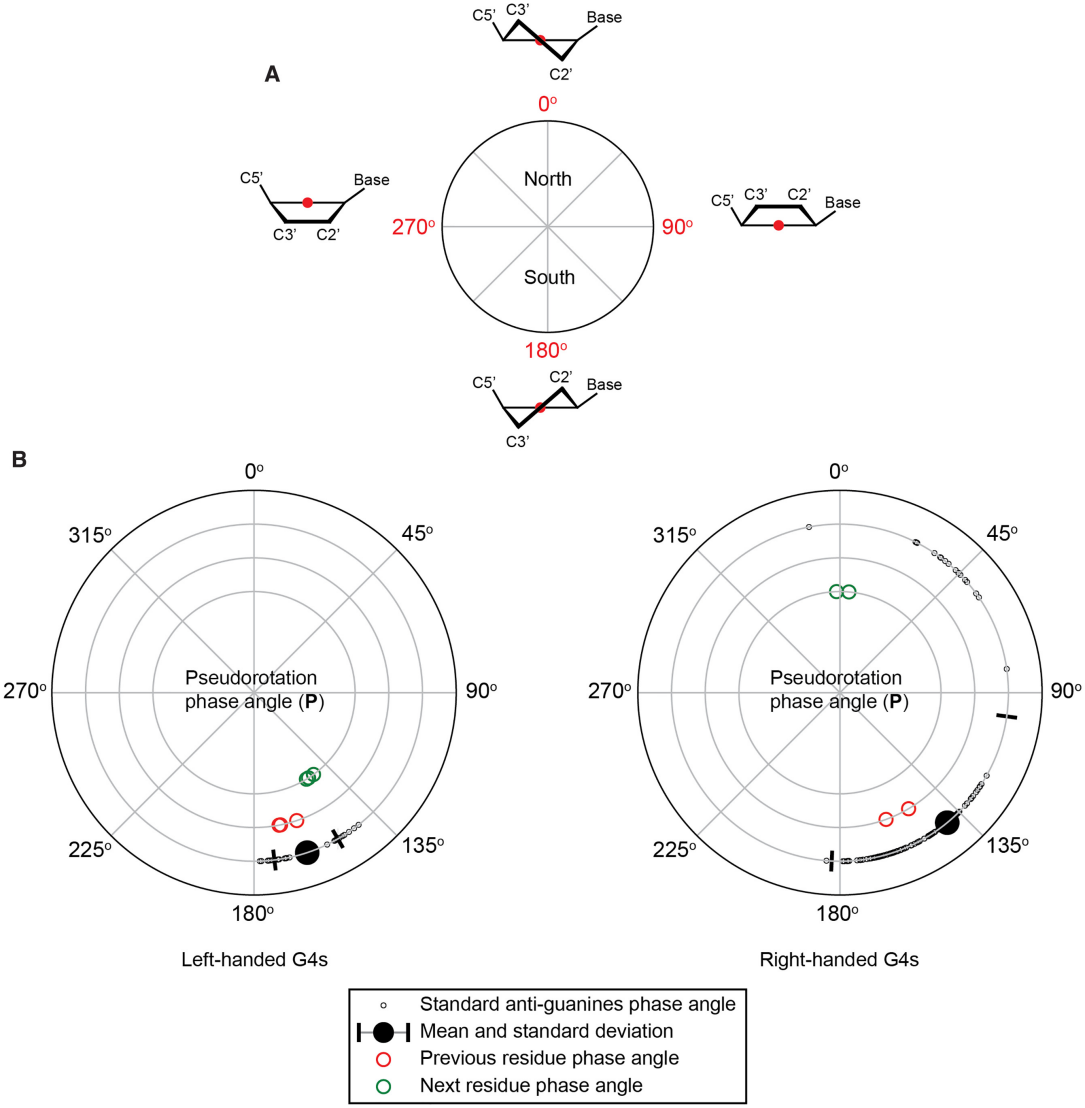
Comparison of pseudorotation phase angles between bulged and non-bulged G4s in left- and right-handed systems. (**A**) Sugar puckering system in terms of pseudorotation phase angle (*P*) (69). (**B**) Comparison diagrams of the left- and right-handed G4 DNA sugar puckering in terms of *P* values. Black circles indicate standard values (PDB ID: 4U5M, 6GZ6, 1KF1, 244D and 352D) (8,46,49,67,68), with the mean and standard deviations denoted; red and green circles indicate the *P* values of the ‘previous residue’ and ‘next residue’ before and after the bulge, respectively (structures of *1xBulge-LHG4motif-TT*,*2xBulge-LHG4motif-TT* and PDB ID: 5UA3) (40).

The presence of a bulge also affects the sugar puckering of the core guanine residues immediately adjacent to the bulge. Here, we quantified the alteration effect of the sugar puckers of the ‘previous residue’ and ‘next residue’ by the standard pseudorotation phase angle parameter (*P*) (Figure 6A) (69). Standard values of *P* were measured using the same set of crystal structures (PDB ID: 4U5M, 6GZ6, 1KF1, 244D and 352D) (8,46,49,67,68) for both left- and right-handed G4 systems. The *P* values were obtained from *1xBulge-LHG4motif-TT* and *2xBulge-LHG4motif-TT* structures for left-handed G4s and the crystal structure 5UA3 (40) for a right-handed G4. The summary of the comparison is presented in Figure 6B.

In non-bulged left-handed G4 systems, all the core guanine residues adopt C2’-*endo* conformations (135° < *P* < 180°). Upon introduction of a bulge, both the ‘previous residue’ and ‘next residue’ adjacent to the bulge do not undergo changes in sugar pucker conformation, staying in C2’-*endo* conformations. Comparatively, in non-bulged right-handed G4 systems, the majority of the core guanine residues similarly adopt C2’-*endo* conformations. However, in the presence of a bulge, the ‘next residue’ sugar pucker conformation was altered to C3’-*endo*, with the *P* values around 0°. This analysis showed that the presence of a bulge in left-handed G4s requires less alteration in terms of sugar puckering compared to a bulge in right-handed G4s.

To complement the pseudorotation phase angle which can be difficult to visualize, we introduced the term *z*-deviation as an alternative parameter which is defined as the perpendicular distance between the plane containing the atoms C4’, O4’ and C1’ to either C2’ or C3’ (Supplementary Figure S14A). Analogous comparisons between non-bulged and bulged left- and right-handed G4s are presented (Supplementary Figure S14B). Like for the *P* values, the only significant change was observed for the C2’ *z*-deviation values of the ‘next residue’ in right-handed bulged G4 which was negative instead of the positive values for the standard G4s. As expected, both the C3’ and C2’ *z*-deviation values of bulged left-handed G4 structures are unchanged from the standard left-handed G4s.

### Bulges and loops in left- and right-handed G4s

Outside of the G-tetrad core formed by four G-columns (or G-tracts), the two significant structural elements of a G4 structure are the loops and bulges. By definition, a loop is a nucleotide chain ranging from zero (only a single phosphate group) to several bases that connects two separate G-columns, while a bulge is a nucleotide chain of at least one base between two stacking guanines in the same G-column. In the context of sequences with alternating guanines and thymines, the concepts of loops and bulges might be undistinguishable based on the sequences alone, whereby the structures are required to recognize them. To do that, the stacking guanines are to be identified first. For example, in left-handed G4s the stacking guanines are defined to have a left-handed helical progression with a base rotation magnitude of ∼27° (46). In this definition, bulges and loops of a G4 structure can be readily distinguished.

This study revealed the detailed similarities and differences between the conformations of single-nucleotide bulges in left- and right-handed parallel G4s. Among the four structural elements (single-nucleotide bulges and loops in left- and right-handed parallel G4s), the loop in left-handed G4 has the most distinct feature allowing to pack a tight T-cap in an inward direction covering the ends of the G-tetrad core, whilst all the other three elements have outward projections (Supplementary Figure S15). Another distinguishing feature is the local sugar orientation (from 5′ to 3′) on each of the elements. Using the crystal structures of a bulged parallel left-handed G4 (*1xBulge-LHG4motif-TT*) and a bulged parallel right-handed G4 (PDB ID: 5UA3), we can see that: the local sugar orientations of the left-handed loop and the right-handed bulge are parallel to (same direction of) those of the G-tetrad guanines; while those of the left-handed bulge and the right-handed loop are anti-parallel to (opposite direction of) those of the G-tetrad guanines (Supplementary Figure S15).

## CONCLUSION

Here we report on the ability of the 12-nt sequence GTGGTGGTGGTG called *LHG4motif* to drive several DNA sequences towards left-handed G4s with bulges. We present the first crystal and NMR solution structures of left-handed G4s containing one, two and three bulges. The bulged residues were found to be pointing outwards of the G-tetrad core in all the structures. Thermal stability and geometrical analyses showed that the presence of bulges in left-handed G4s is associated with less disturbing and penalizing effect when compared to the right-handed counterparts.

## DATA AVAILABILITY

The coordinates for the X-ray crystal structures of two left-handed G4s containing one and two bulges and the NMR solution structures of a left-handed G4 containing three bulges have been deposited in the Protein Data Bank (PDB codes: 7D5D, 7D5E and 7D5F).

## Supplementary Material

gkaa1259_Supplemental_File
