# Supplementary material for: Bulges in left-handed G-quadruplexes
Source: Nucleic Acids Res. 2021 Jan 27;49(3):1724–36. doi: 10.1093/nar/gkaa1259 (PMC7897477; doi:10.1093/nar/gkaa1259)
Supplement: gkaa1259_Supplemental_File [file gkaa1259_supplemental_file.docx]

**Bulges in left-handed G-quadruplexes**

Poulomi Das,^1^ Khac Huy Ngo,^1^ Fernaldo Richtia Winnerdy,^1^ Arijit Maity,^1^ Blaž Bakalar,^1^ Yves Mechulam,^2^ Emmanuelle Schmitt ^2^ and Anh Tuân Phan^1,3,^*

^1^ School of Physical and Mathematical Sciences, Nanyang Technological University, Singapore 637371, Singapore

^2^ Laboratoire de Biochimie, UMR 7654, CNRS, Ecole Polytechnique, Palaiseau 91128, France

^3^ NTU Institute of Structural Biology, Nanyang Technological University, Singapore 636921, Singapore

**Supplementary Information**

**Table S1**: List of designed potential bulge-containing motifs to be attached with *LHG4motif*

| **Name** | **Sequence (5’-3’)** |
| --- | --- |
| ***1xBulge*** | GGTG**T**GTGGTGG |
| ***2xBulge*** | GGTG**T**GTG**T**GTGG |
| ***3xBulge*** | GGTG**T**GTG**T**GTG**T**G |
| ***4xBulge*** | G**T**GTG**T**GTG**T**GTG**T**G |

**Table S2**: List of sequences with *LHG4motif* being attached at the 5’-end

| **Name** | **Sequence (5’-3’)** |
| --- | --- |
| ***LHG4motif-1xBulge*** | **GTGGTGGTGGTG** T GGTG**T**GTGGTGG |
| ***LHG4motif-2xBulge*** | **GTGGTGGTGGTG** T GGTG**T**GTG**T**GTGG |
| ***LHG4motif-3xBulge*** | **GTGGTGGTGGTG** T GGTG**T**GTG**T**GTG**T**G |

**Table S3**: List of sequences used in crystallization with additional two thymines at 3’-end (in bold)

| **Name** | **Sequence (5’-3’)** |
| --- | --- |
| ***1xBulge-LHG4motif-TT*** | GGTG**T**GTGGTGG T GTGGTGGTGGTG **TT** |
| ***2xBulge-LHG4motif-TT*** | GGTG**T**GTG**T**GTGG T GTGGTGGTGGTG **TT** |

**Table S4**: Comparison of destabilization effect caused by addition of bulges in right-handed and left-handed G4s.

| **Type of G4s** | **T_m_ (°C)** | **ΔT_m_ (°C)^a^** |
| --- | --- | --- |
| Right-handed (in 60 mM K^+^) | | |
| One bulge | 77.6 ± 3.1^b^ | - |
| Two bulges | 60.2 ± 4.1^b^ | 17.5 ± 5.1 |
| Three bulges | 39.0 ± 9.4^b^ | 21.2 ± 9.9 |
| Four bulges | N/A | N/A |
| Left-handed (in 100 mM K^+^) | | |
| One bulge | 59.6 ± 0.6 | - |
| Two bulges | 43.5 ± 0.6 | 16.1 ± 0.6 |
| Three bulges | 31.4 ± 0.6 | 12.0 ± 0.6 |
| Four bulges | N/A | N/A |

^a^ The ΔT_m_ are the melting temperature differences upon additions of the latest bulge residues.

^b^ The values for one, two and three bulges are averages of the measured melting temperatures of eight, six and four sequences, respectively.


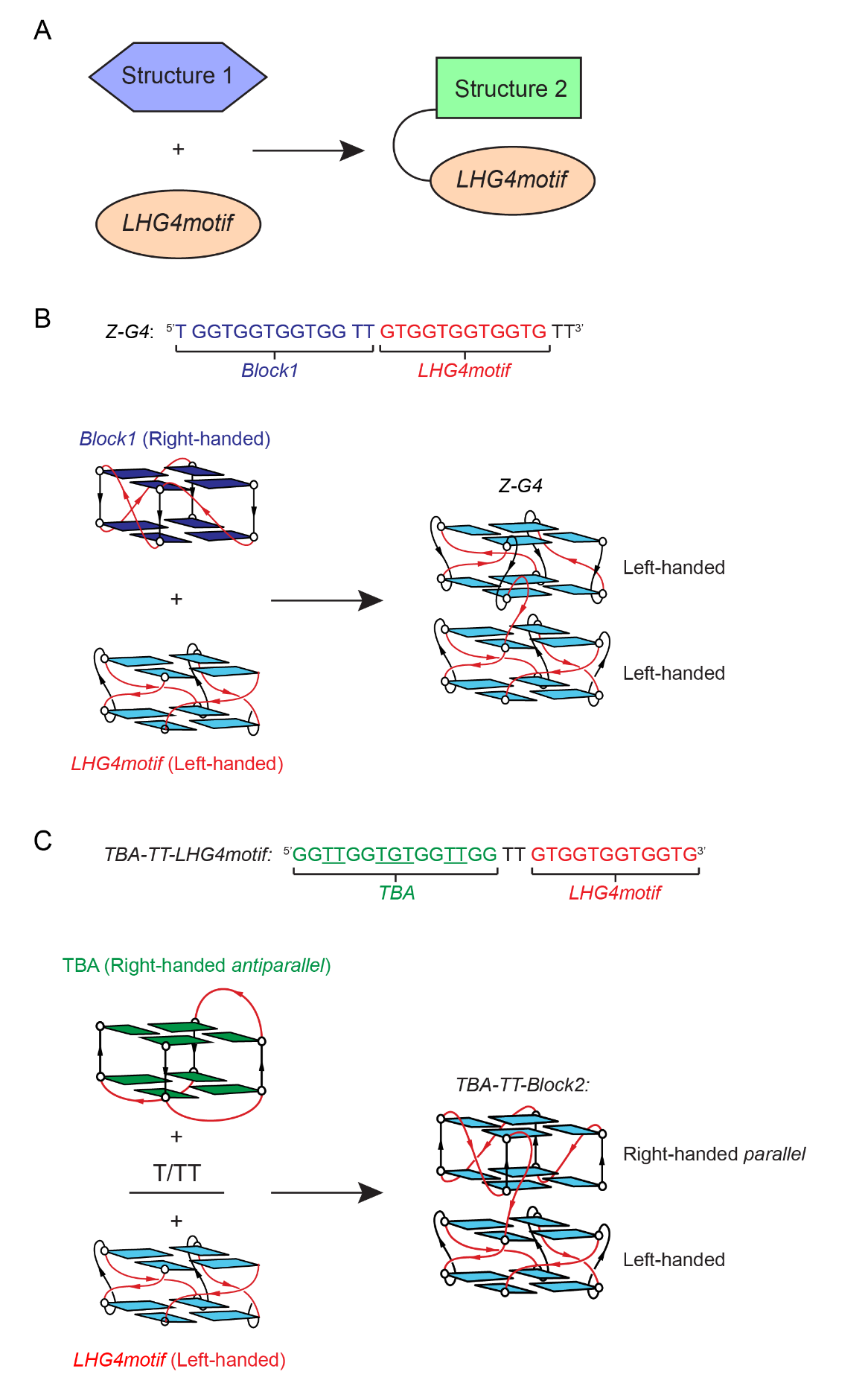


**Figure S1**: **(A)** Schematics showing the possible structural transformation of DNA sequences on merging with *LHG4motif*. The label ‘Structure 1’ in the schematic may include stable G4 structures or unstructured DNA chains. Schematic representation of structural transformation of **(B)** *Block1* from right-handed G4 to left-handed G4 when attached with *LHG4motif* (1) **(C)** *TBA* from right-handed antiparallel G4 to right-handed parallel on merging with *LHG4motif* (2).

**
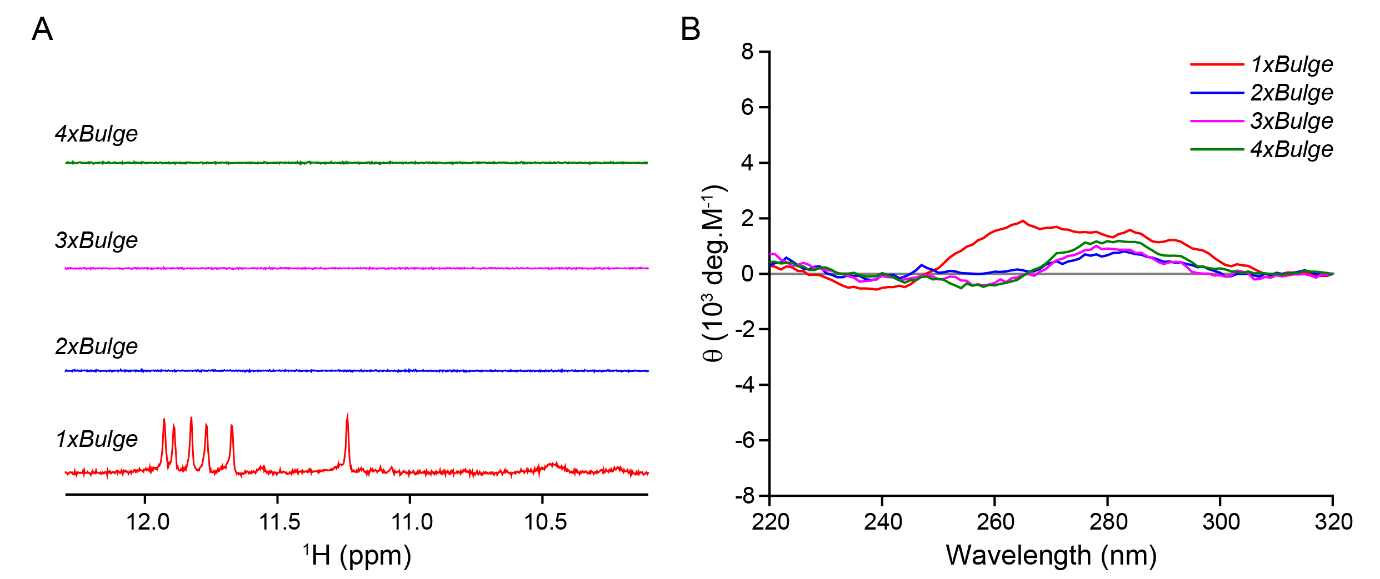
**

**Figure S2**: **(A)** 1D ^1^H NMR and **(B)** CD spectra of the designed sequences listed in Table S1 which forms unknown or no structure independently.


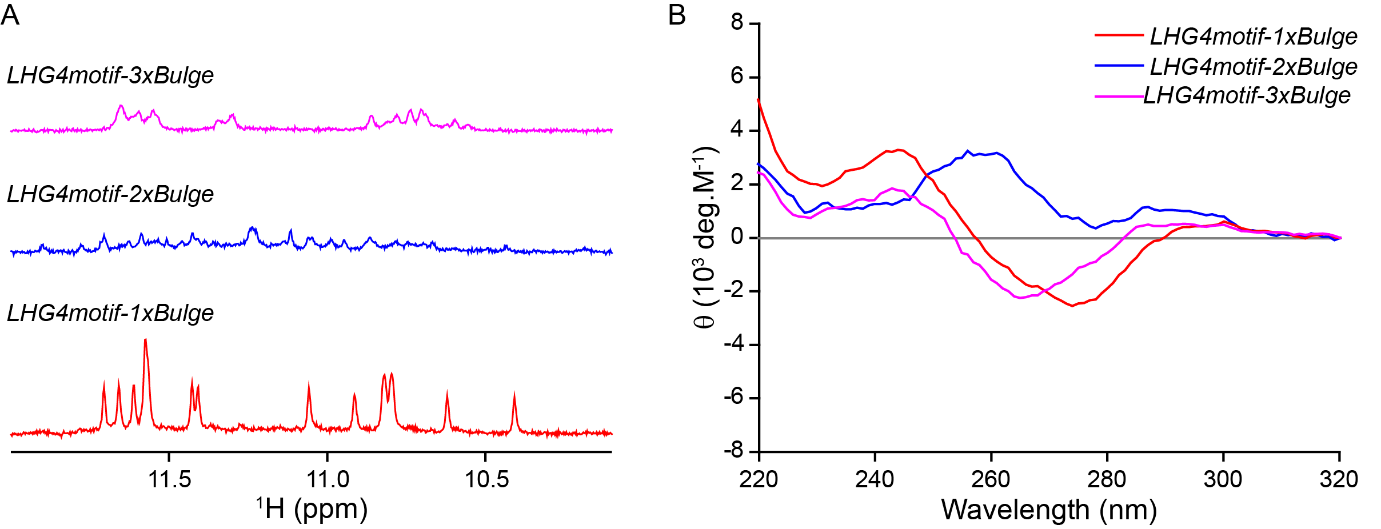


**Figure S3: (A)** 1D ^1^H NMR and **(B)** CD spectra of the designed sequences when *LHG4motif* is attached to its 5’-end as listed in Table S2.


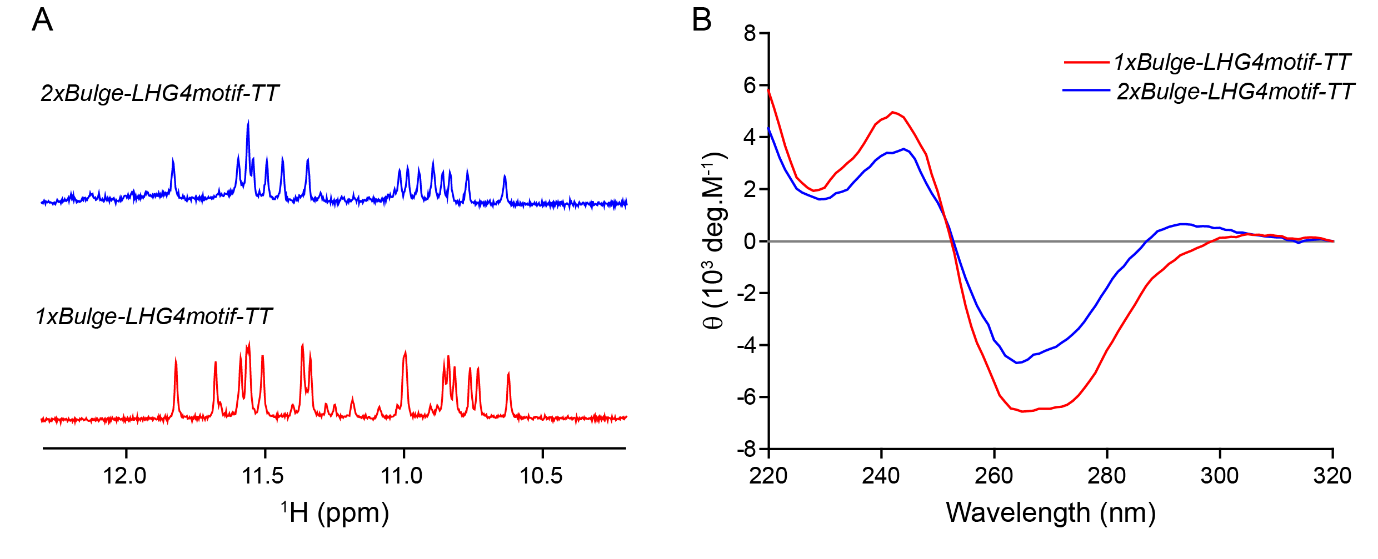


**Figure S4:** **(A)** 1D NMR and **(B)** Circular dichroism (CD) spectra of left-handed g-quadruplexes containing one (*1xBulge-LHG4motif-TT*; red), two (*2xBulge-LHG4motif-TT*; blue), bulges. The distinct characteristics of left-handed g-quadruplexes are clearly observed in NMR spectra displaying sixteen peaks divided into two groups of eight peaks and the molar ellipticity ($\theta$) in the CD spectra possessing crest and trough at 240 nm and 265 nm respectively.


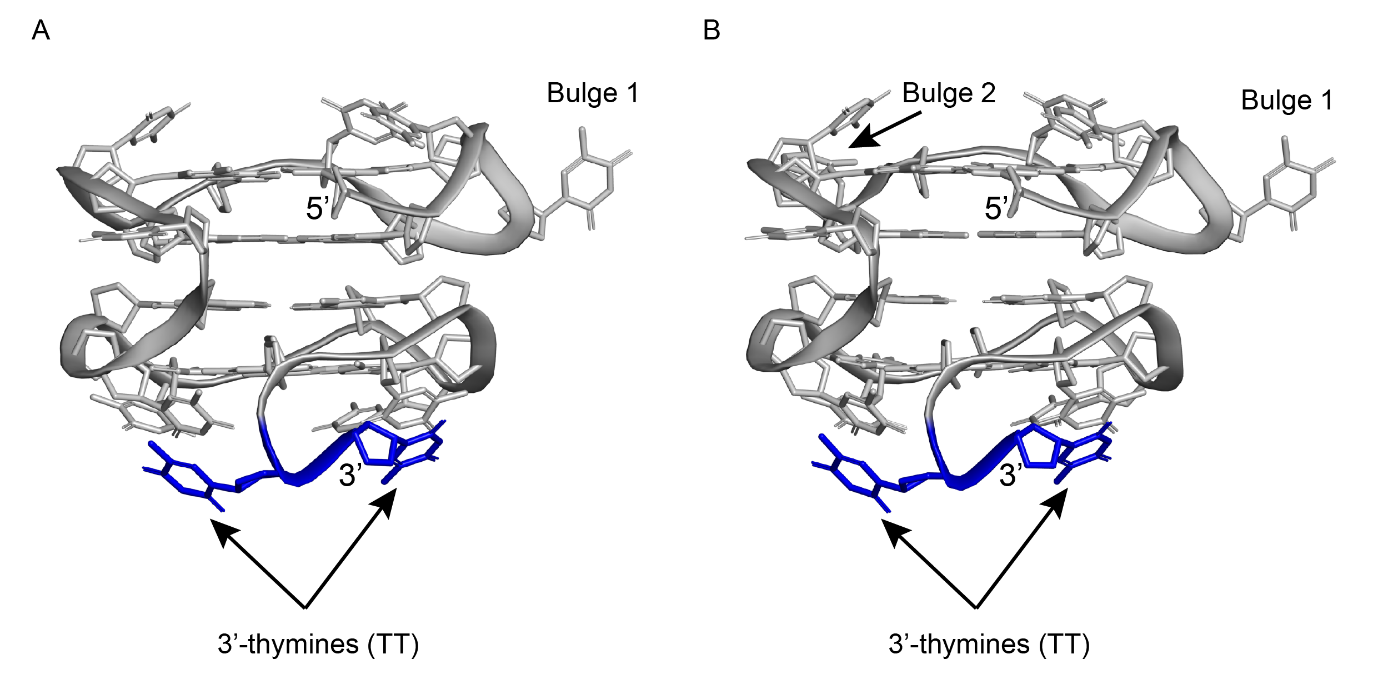


**Figure S5:** Ribbon view of **(A)** *1xBulge-LHG4motif-TT* and **(B)** *2xBulge-LHG4motif-TT* structures highlighting the 3’-end stacking thymines (TT) in blue.


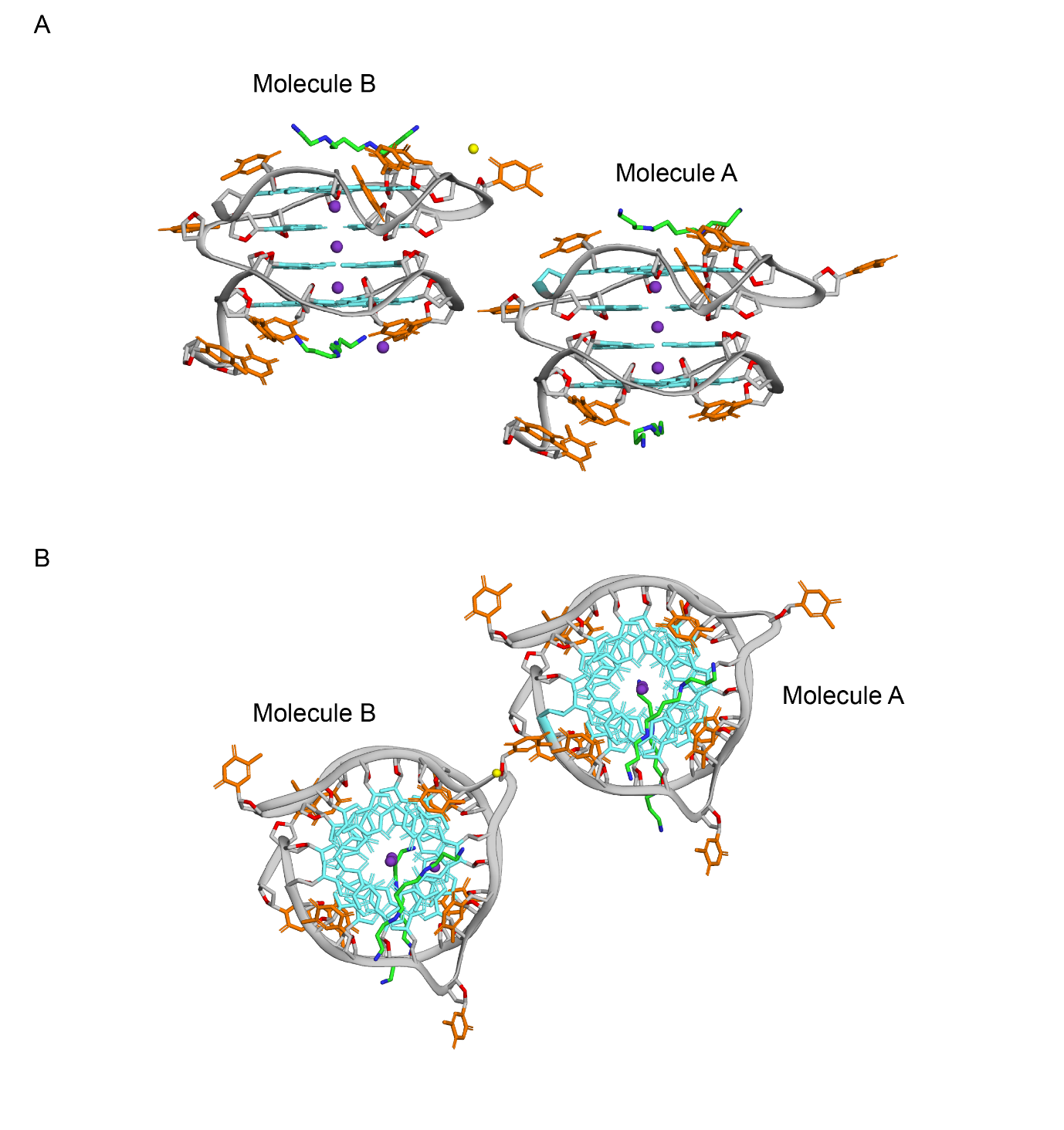


**Figure S6: (A)** Side view and **(B)** top-view of two crystal structures of *2xBulge-LHG4motif-TT* named as Molecule A and Molecule B found in a unit cell. Potassium ions, sodium ions, spermine carbon and nitrogen atoms are shown in purple, yellow, green and blue respectively.

**
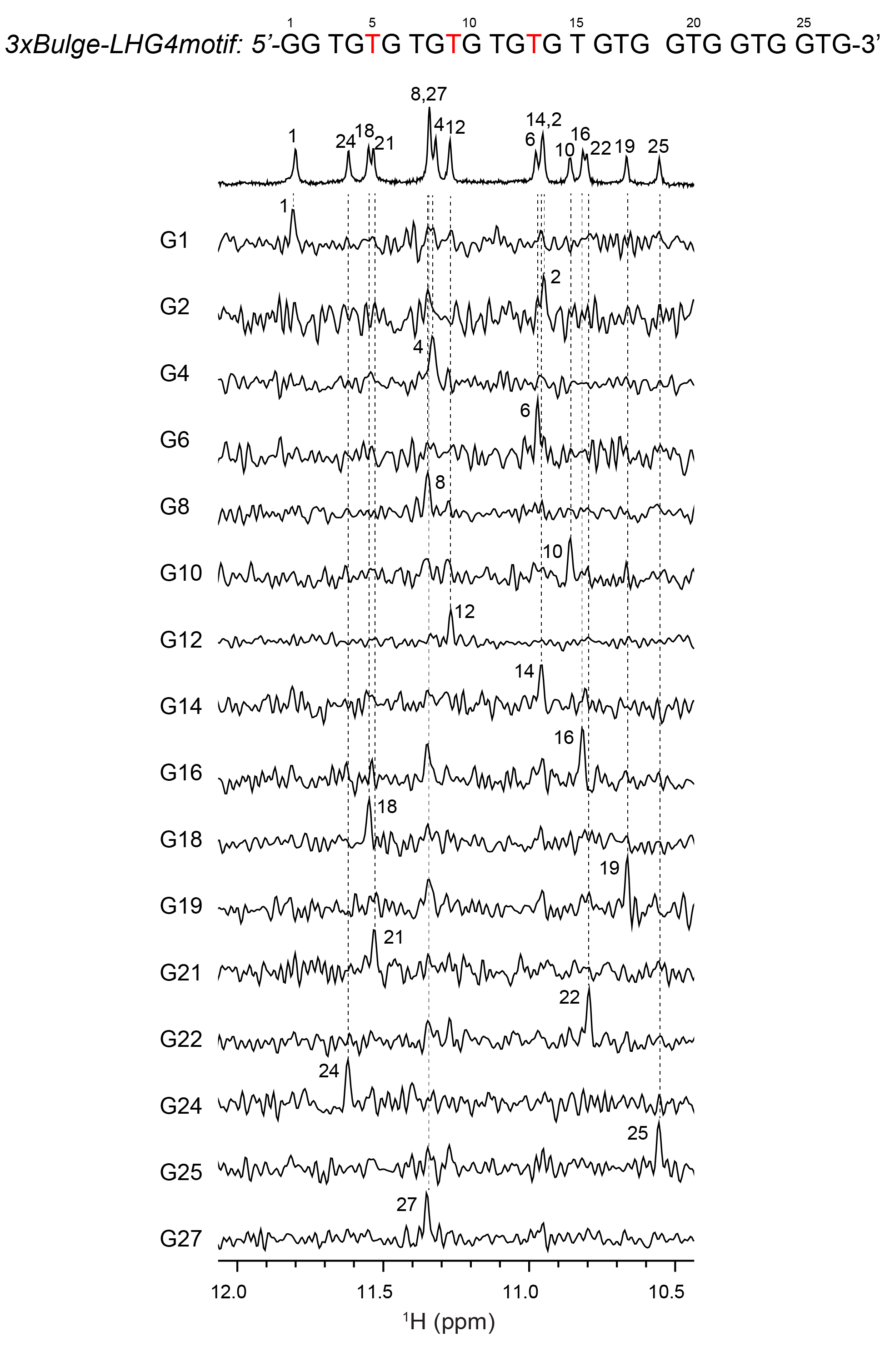
**

**Figure S7:** Unambiguous assignment of guanine imino proton using site specific 2% ^15^N-labelling.

**
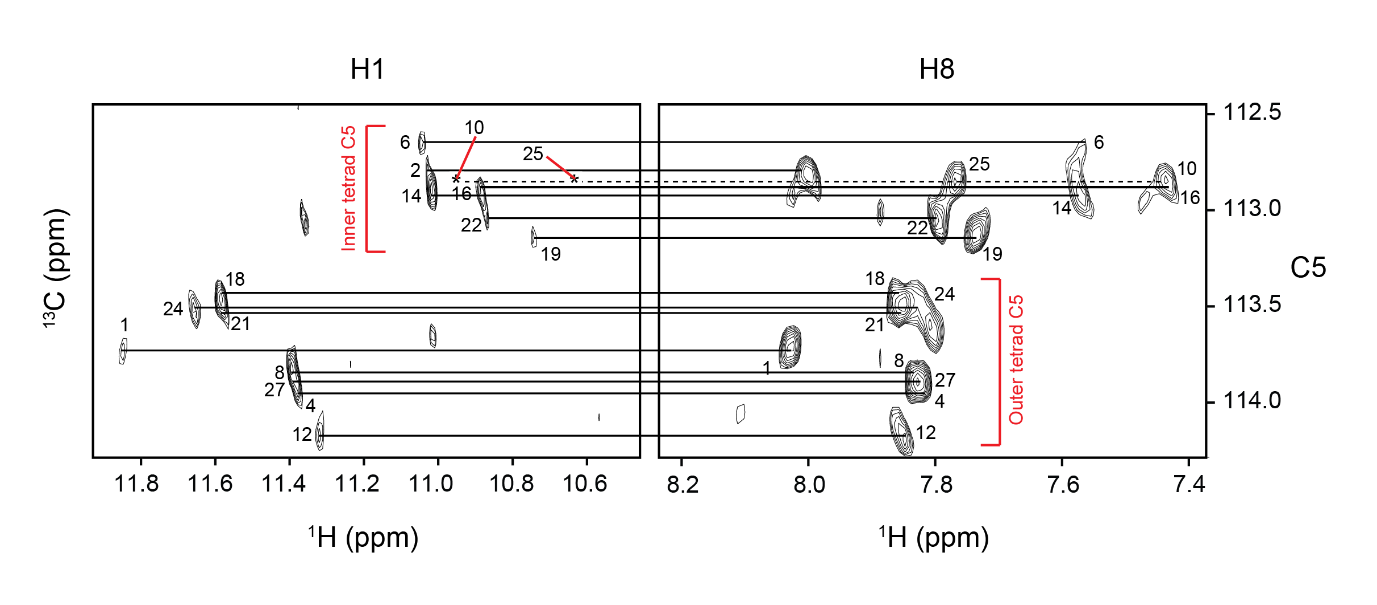
Figure S8:** Spectral assignments of guanine aromatic (H8) protons from long range through-bond correlations between guanine imino H1 protons and aromatic H8 protons via ^13^C5. Missing cross-peaks (too low intensity) are indicated by asterisks (*).


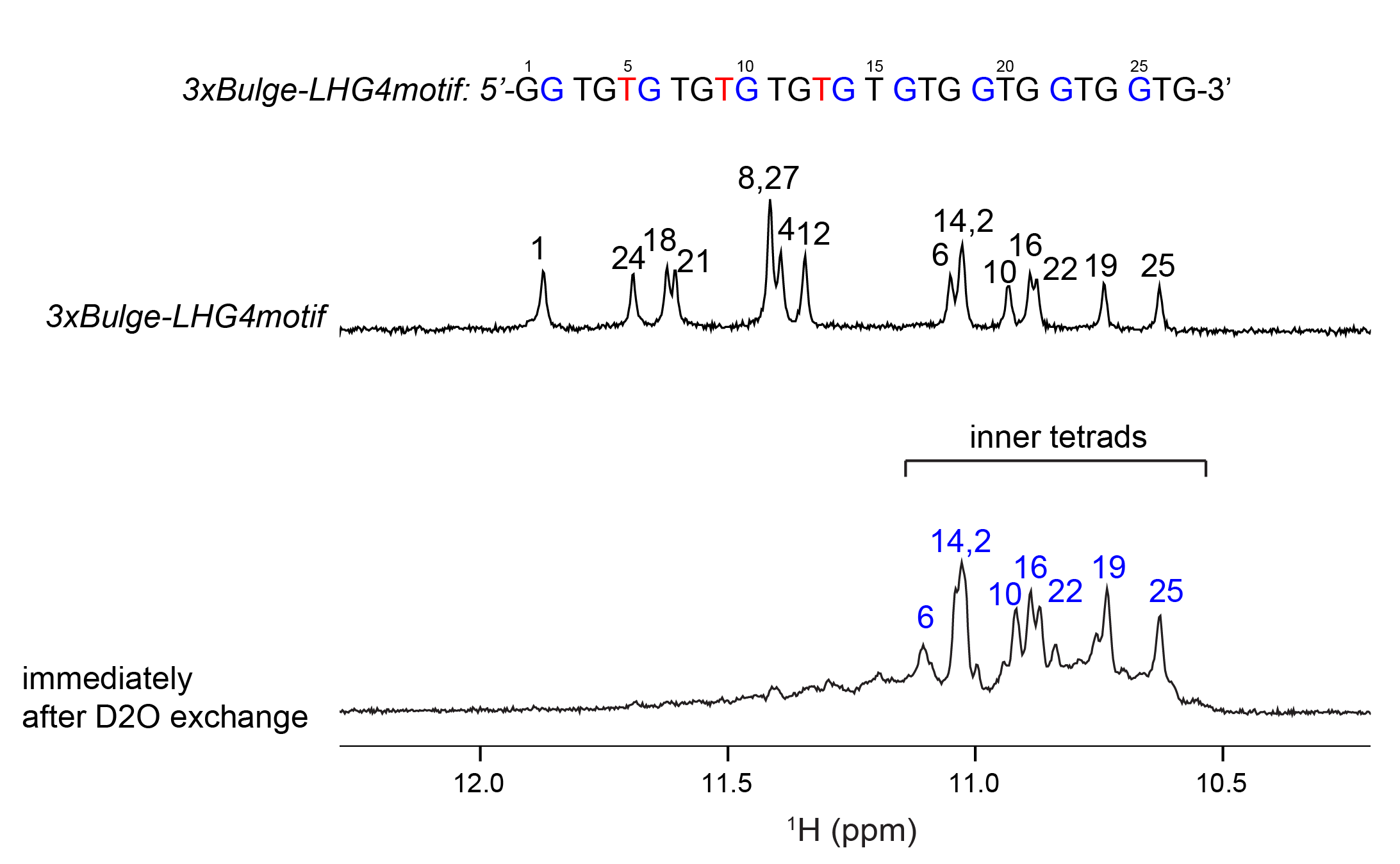


**Figure S9:** D_2_O solvent exchange of the *3xBulge-LHG4motif* sample. The bottom spectrum was recorded immediately after adding 100% D_2_O. The guanine imino protons assigned in the bottom spectra correspond to the guanines forming the inner tetrad which are protected from solvent exchange.


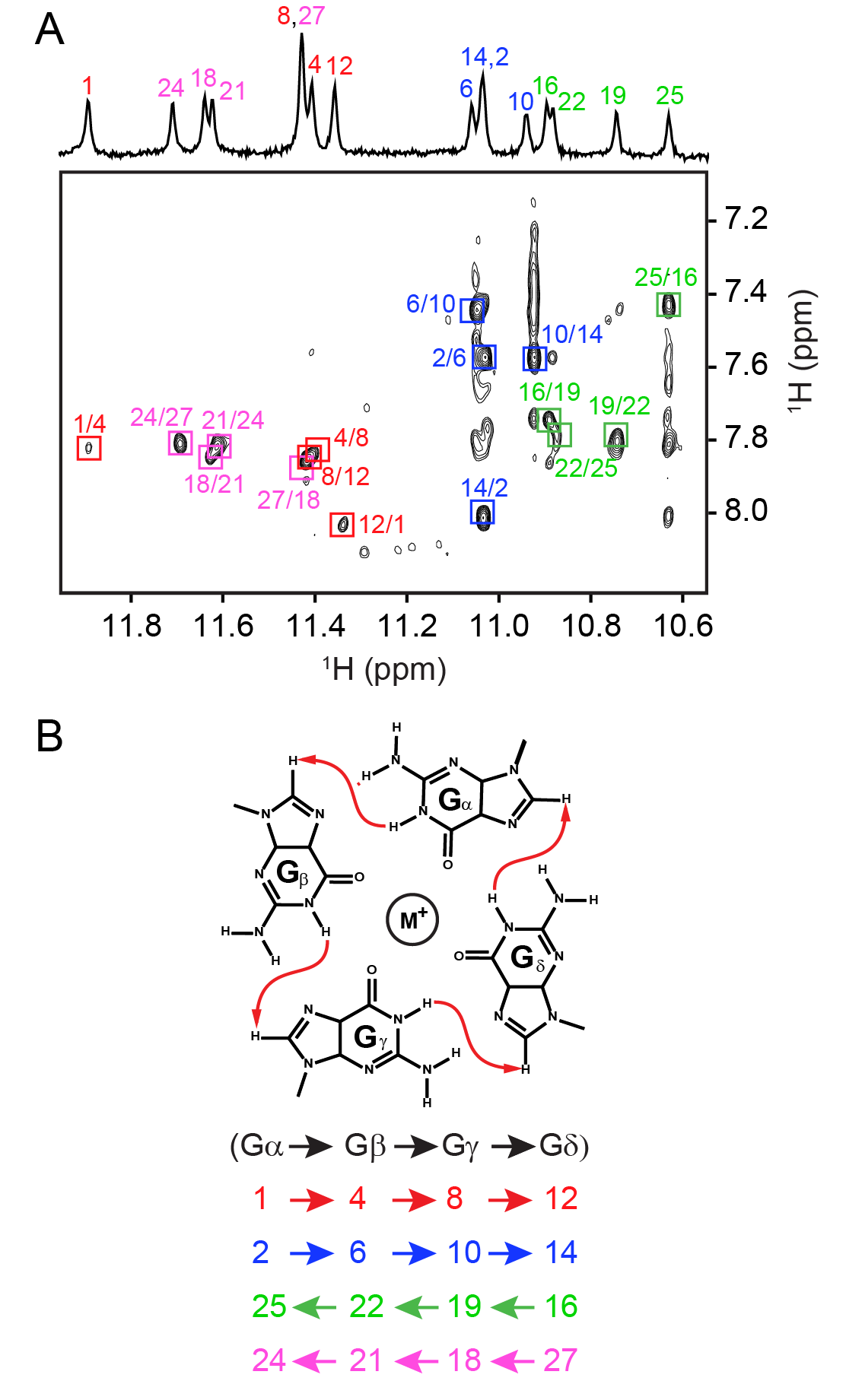


**Figure S10: (A)** NOESY spectrum of *3xBulge-LHG4motif* with mixing time 200 ms recorded in presence of 106 mM K^+^ in H_2_O showing guanine imino (H1)-aromatic (H8) cyclic-connectivity patterns in same tetrad. Four G-tetrads are marked with different colors displaying the H1-H8 cross-peaks which are labeled with the sequence numbers **(B)** Schematic representation of G-tetrad NOE connectivity pattern and cyclic orientations of guanines from each G-tetrads.


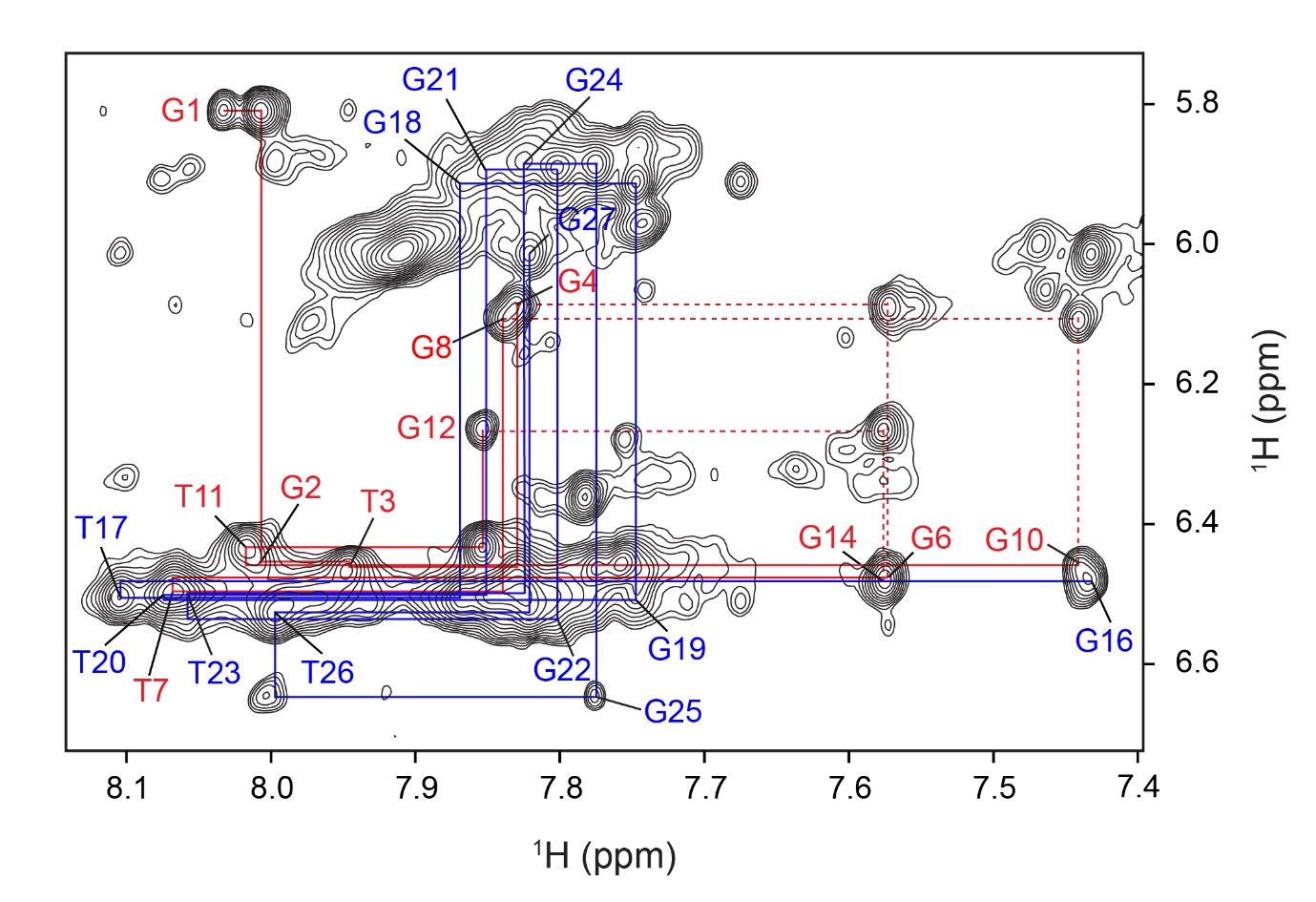


**Figure S11:** D_2_O NOESY spectrum (mixing time, 300 ms) of *3xBulge-LHG4motif* at 25°C in H8/H6-H1’ region. Modified sequential H8_(n)_-H1’_(n)_-H8_(n+1/n+2)_ walk of the two blocks are shown in red and blue respectively. Intra residue H8-H1’ cross-peaks are marked with the corresponding residue number. Traditional sequential walk is indicated with solid lines for the whole sequence except the part surrounding thymine bulges and linker (T5, T9, T13 and T15). For the bulges, we indicated the stacking correlation between G4-to-G6, G8-to-G10 and G12-to-G14 with dashed lines, skipping the bulge residues T5, T9 and T13 due to non-existence of sequential inter-residue cross-peaks. The connection to the linker T15 is skipped entirely due to the same reason.


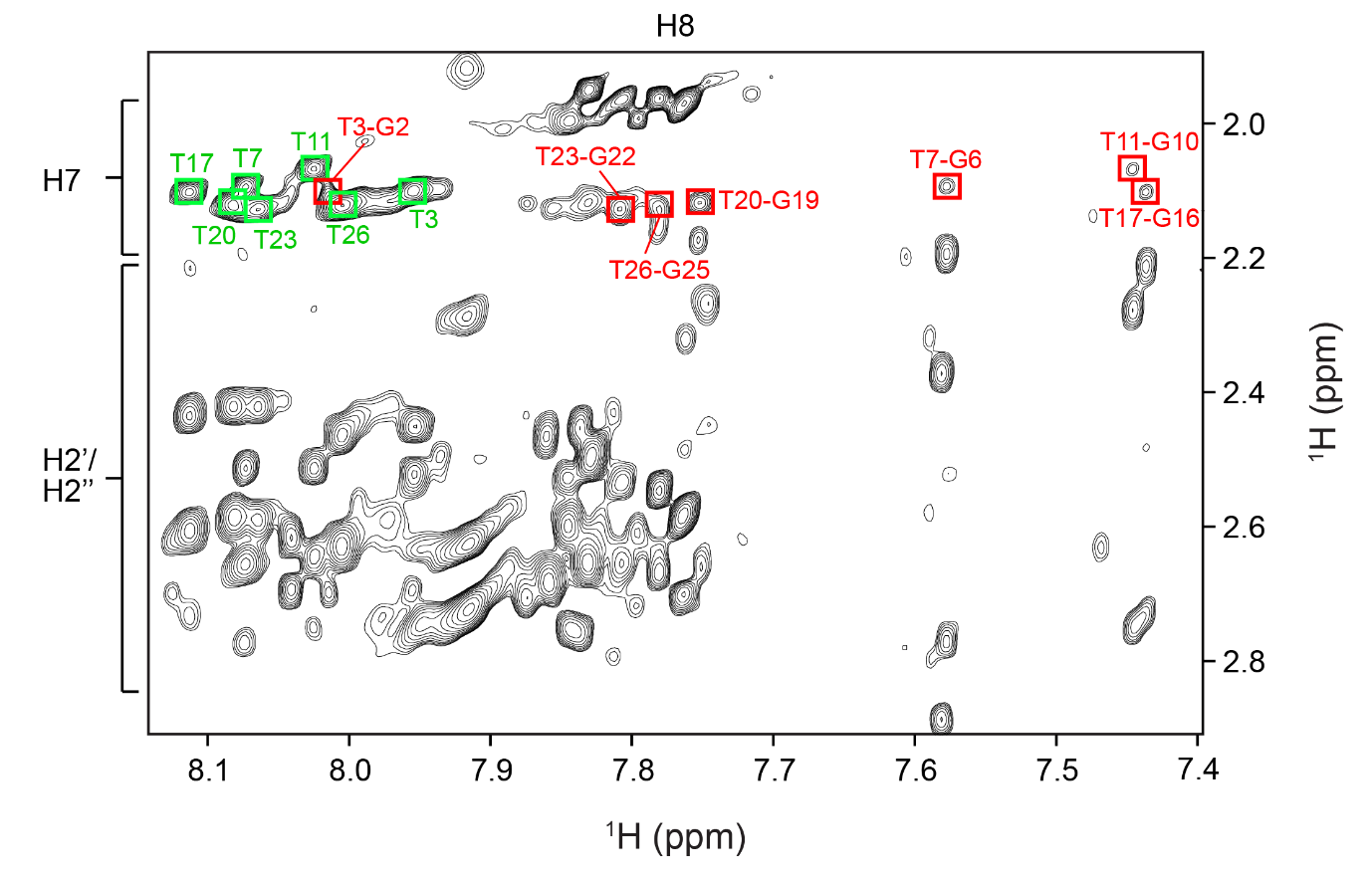


**Figure S12:** D_2_O NOESY spectrum (mixing time, 300 ms) of *3xBulge-LHG4motif* at 25°C in H8/H6-H2’/H2’’/H7 region. The spectrum showed sharp cross-peaks between H8/H6 and H2’/H2’’ that were used to assist the assignments of aromatic peaks. Indicated in green boxes are the intra-residue cross peaks of capping thymines between the aromatic protons H6 and the methyl protons H71/72/73. Shown in red boxes are the inter-residue cross peaks between the aromatic protons (H8) of the outer tetrad guanines (G2, G6, G10, G16, G19, G22 and G25) and the methyl protons (H71/72/73) of the capping thymines (T3, T7, T11, T17, T20, T23 and T26). These cross peaks support the formation of the capping features of the G4 structure.


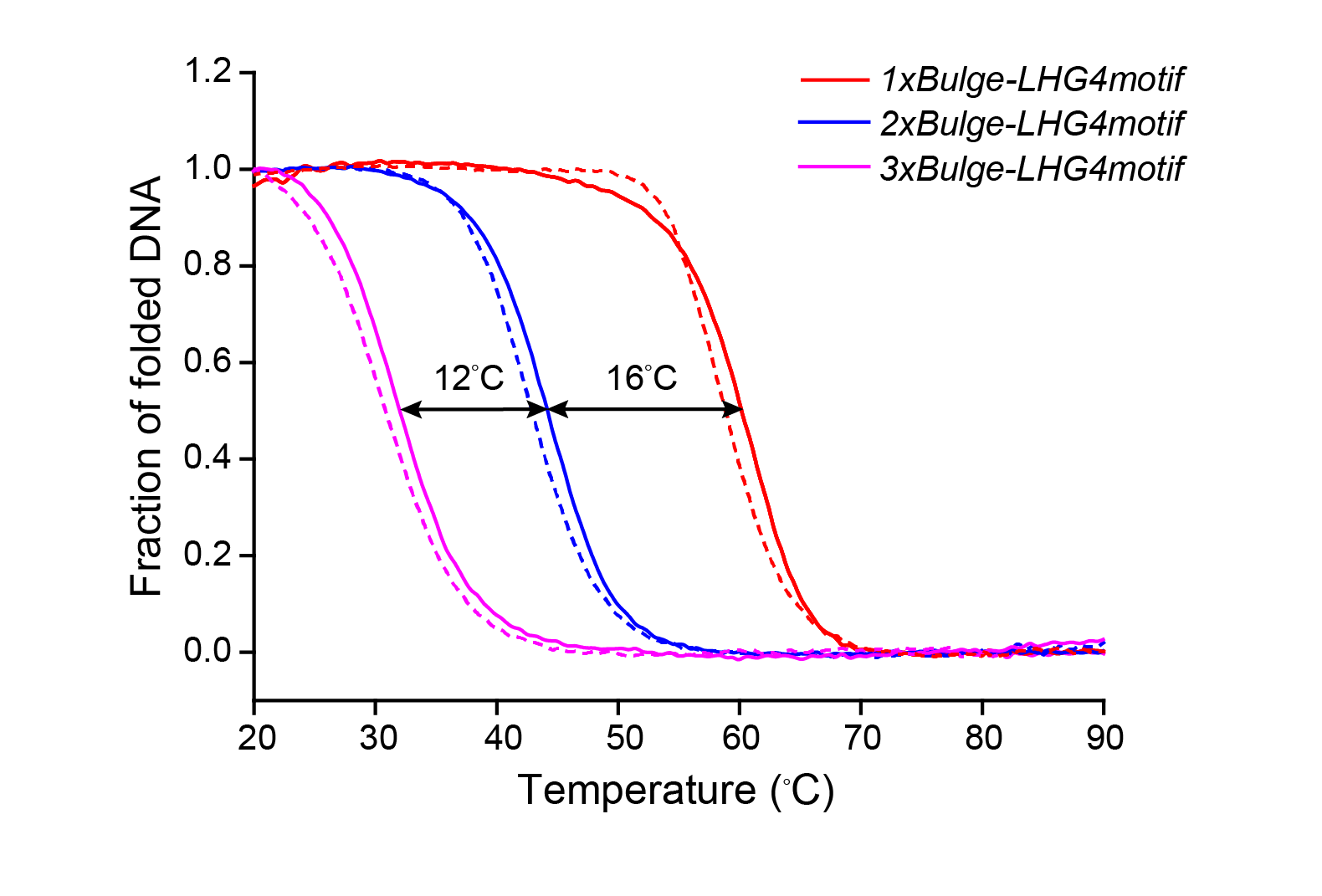


**Figure S13:** UV melting curves showing the decrease in melting point (T_m_) with increased number of bulges. Solid and dashed lines indicate heating and cooling curves respectively.


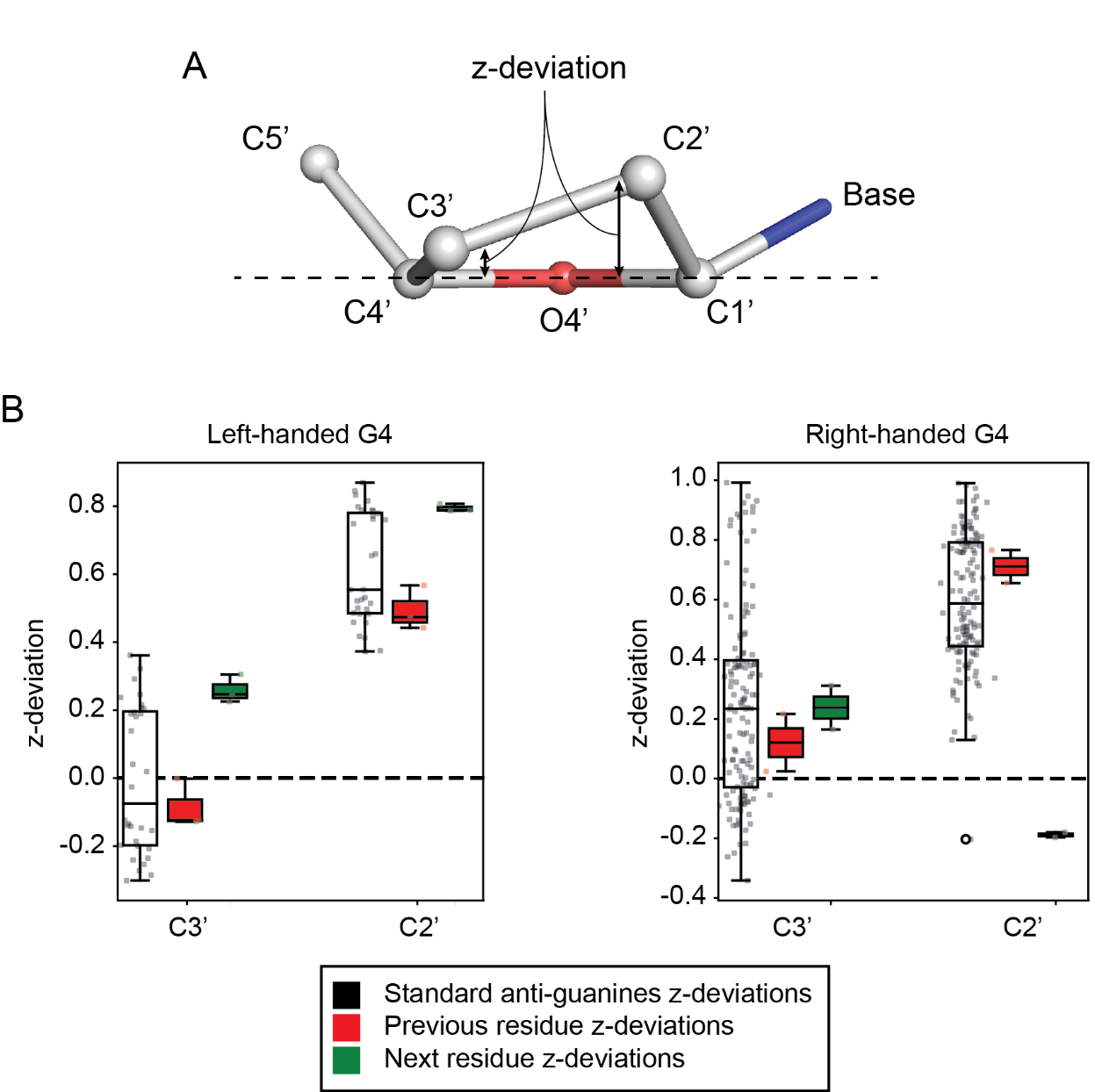


**Figure S14:** Statistical comparison of sugar pucker conformations between bulged vs non-bulged G4s in left- and right-handed systems. (A) Schematic of the z-deviations of C2’ and C3’. The dashed line indicates a plane comprised of the atoms C4’, O4’ and C1’. (B) Boxplot diagrams of left- and right-handed G4s. White boxes represent the z-deviation distributions of standard G4s (PDB ID: 4U5M, 6GZ6, 1KF1, 244D and 352D) (1,3-6); red boxes indicate the values for “previous residue”; green boxed indicate the values for the “next residue” (structures of *1xBulge-LHG4motif, 2xBulge-LHG4motif* and PDB ID: 5UA3) (7).

**
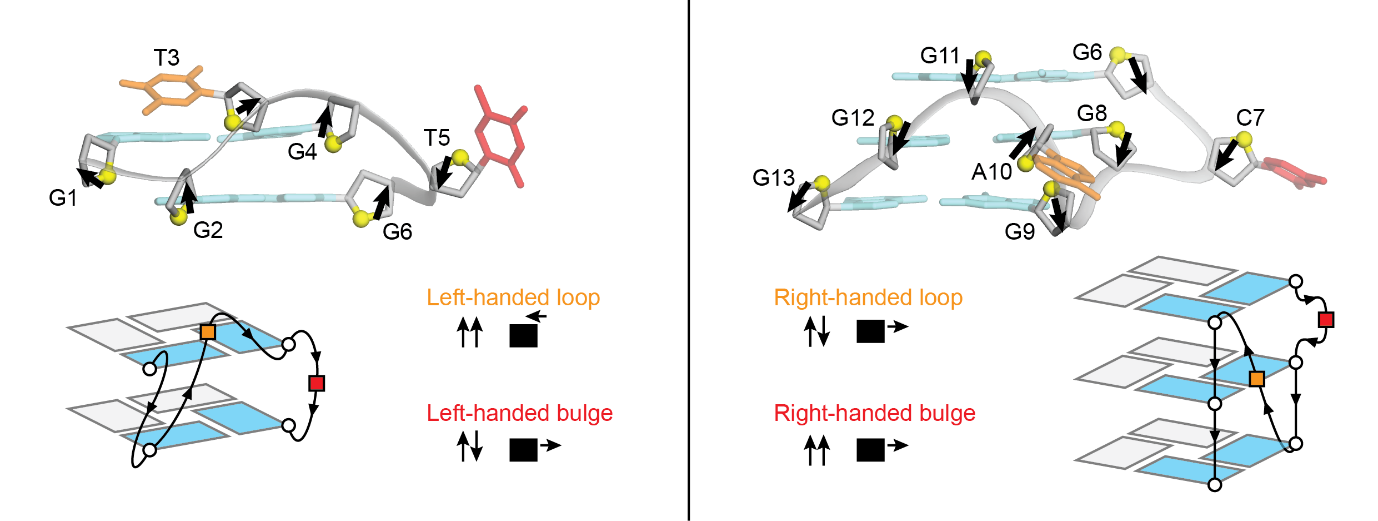
**

**Figure S15:** Structures and schematic representations of a bulge-containing left-handed G4 (*1xBulge-LHG4motif*) and a bulge-containing right-handed G4 (PDB ID: 5UA3). Cyan color indicates the G-tetrad guanines, while the orange and red colors indicate the loops and bulges respectively. The thick arrows in the structure diagrams indicate the local sugar orientations of each nucleotide (from 5’ to 3’). The double-arrow symbols indicate “parallel” (less that 90^o^ difference) and “anti-parallel” (more than 90^o^ difference) relative local sugar orientations between the G-tetrad guanines and the loops or bulges. The box-with-arrow symbols indicate the inward and outward projections of the base of the loops or bulges.

**References**

1. Chung, W.J., Heddi, B., Schmitt, E., Lim, K.W., Mechulam, Y. and Phan, A.T. (2015) Structure of a left-handed DNA G-quadruplex. *Proc Natl Acad Sci U S A*, **112**, 2729-2733.

2. Winnerdy, F.R., Bakalar, B., Maity, A., Vandana, J.J., Mechulam, Y., Schmitt, E. and Phan, A.T. (2019) NMR solution and X-ray crystal structures of a DNA molecule containing both right- and left-handed parallel-stranded G-quadruplexes. *Nucleic Acids Res*, **47**, 8272-8281.

3. Bakalar, B., Heddi, B., Schmitt, E., Mechulam, Y. and Phan, A.T. (2019) A Minimal Sequence for Left-Handed G-Quadruplex Formation. *Angew Chem Int Ed Engl*, **58**, 2331-2335.

4. Laughlan, G., Murchie, A.I., Norman, D.G., Moore, M.H., Moody, P.C., Lilley, D.M. and Luisi, B. (1994) The high-resolution crystal structure of a parallel-stranded guanine tetraplex. *Science*, **265**, 520-524.

5. Phillips, K., Dauter, Z., Murchie, A.I., Lilley, D.M. and Luisi, B. (1997) The crystal structure of a parallel-stranded guanine tetraplex at 0.95 A resolution. *J Mol Biol*, **273**, 171-182.

6. Parkinson, G.N., Lee, M.P. and Neidle, S. (2002) Crystal structure of parallel quadruplexes from human telomeric DNA. *Nature*, **417**, 876-880.

7. Meier, M., Moya-Torres, A., Krahn, N.J., McDougall, M.D., Orriss, G.L., McRae, E.K.S., Booy, E.P., McEleney, K., Patel, T.R., McKenna, S.A. *et al.* (2018) Structure and hydrodynamics of a DNA G-quadruplex with a cytosine bulge. *Nucleic Acids Res*, **46**, 5319-5331.
